# Computational Design of Lysine Targeting Covalent Binders Using Rosetta

**DOI:** 10.1021/acs.jcim.5c00212

**Published:** 2025-05-29

**Authors:** Barr Tivon, Jan Wiese, Matthias P. Müller, Ronen Gabizon, Daniel Rauh, Nir London

**Affiliations:** † Department of Chemical and Structural Biology, 34976 The Weizmann Institute of Science, Rehovot 7610001, Israel; ‡ Department of Chemistry and Chemical Biology, TU Dortmund University and Drug Discovery Hub Dortmund (DDHD), Zentrum für Integrierte Wirkstoffforschung (ZIW), Otto-Hahn-Strasse 4a, Dortmund 44227, Germany

## Abstract

Chemical probes that form a covalent bond with their target protein have been established as a powerful tool for investigating proteins and modulating their activity, but until recently were mostly targeting cysteine residues. Covalent binders that target lysine residues are increasingly reported. Covalent binding to lysine involves challenges such as the increased p*K*_a_ of the side chain and its considerable flexibility. Here, we describe two computational methods to derivatize lysine-binding covalent small-molecules based on known noncovalent binders, approaching the design problem from two opposite directions. In a “ligand-side” approach, we scan different ligand positions to install an electrophile and dock these derivatized ligands into the target protein. In a “protein-side” approach, we install an electrophile on the target lysine and model its conformational space to find suitable installation vectors on the ligand. We applied both of these protocols retrospectively to a data set of electrophilic ligands and to a data set of vitamin B6 covalently bound to a receptor lysine residue. Our ligand-side protocol successfully identified the known covalent binder in 80% and 86% of cases, while the protein-side protocol achieved identification rates of 56% and 82%, respectively. We prospectively validated these protocols by designing and testing a new lysine-targeting MKK7 inhibitor. Mass-spectrometry and crystallography validated the covalent binding to the target lysine. Applying these protocols to a data set of known kinase inhibitors identified high-confidence covalent candidates for more than 200 human kinases, demonstrating the potential impact of our protocols.

## Graphical abstract

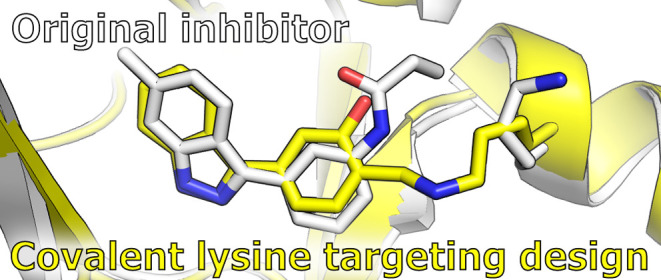

## Introduction

Chemical probes that form a covalent bond with their target protein can offer many advantages over noncovalent probes, such as improved potency, longer duration of action and enhanced selectivity when targeting a nonconserved protein nucleophile.^1–4^ Therefore, there has been an increasing interest in the development of covalent chemical probes and drugs over the past decade, as demonstrated by the large number of covalent compounds that have been reported for a variety of applications and protein targets.

The vast majority of the reported covalent binders have been designed to target a cysteine residue in the target protein^5^. The unique nucleophilicity of cysteines and their relatively low abundance offer great opportunities to design potent and selective covalent binders for many targets in the cellular proteome. However, their low abundance, as well as the oxidizing environment in the extracellular space in which most cysteines exist in disulfide-bonded form, also limit the opportunities in many other cases, where there is no free cysteine near a target binding site. Therefore, efforts have been made to develop novel electrophilic warheads that target other nucleophilic amino acids.^5–10^

Lysine in particular draws much interest, perhaps due to its abundance and nucleophilic nature. Several electrophiles were developed to form a covalent bond with a lysine side-chain^11,12^ including activated esters,^13^ Michael acceptors^14^ and chlorotriazines.^15^ Two electrophile classes stand out as the most widely used: (1) sulfur-fluoride based electrophiles including fluorosulfates^16^ and sulfonyl fluorides.^17^ (2) Benzaldehydes^18^ and their derivatives such as 2-ethynylbenzaldehydes,^19^ benzaldehyde boronic acids,^20,21^ and in particular salicylic aldehydes.^22^

Several covalent probes that target lysines have recently been reported against targets such as TTR,^23,24^ PI3Kδ,^25,26^ BCL-xL,^27^ XIAP,^28^ BCR-ABL,^29^ ALKBH5,^30^ HSP90,^31,32^ eIF4E,^33^ AURKA,^34^ BTK,^35^ 14–3–3 ^36^ and AKT^E17K^,^37^ demonstrating the breadth of protein targets. Covalent lysine targeting was also leveraged for targeted protein degraders,^38^ and efforts were made to proteomically ‘mine’ ligandable lysines.^39–41^

Historically, the most widespread approach to designing covalent binders has relied on the addition of an electrophile to an already-known noncovalent binder that binds near a nucleophilic residue in the target protein.^37,42–48^ Starting from a known noncovalent binder whose potency and selectivity had already been optimized, may reduce the time and effort needed for the development of the covalent binder, and enable the use of a less reactive electrophile, thus improving its selectivity. More recent methodologies include empirical screening of covalent fragment libraries^49–54^ and covalent virtual screening,^55–58^ however, while these methods are able to discover new hits, the typical binding affinities of these initial hits are relatively low, and they require extensive development to reach suitable potency and selectivity.

Identifying the optimal position and choice of an electrophile to install on a known noncovalent binder is not an easy task, as there are numerous possible choices. Therefore, a computational tool that identifies the most promising candidates and reduces the space of possibilities can largely facilitate the design of new covalent binders. Our lab has previously reported Covalentizer,^59^ a computational pipeline that addresses this challenge by creating a virtual library of potential cysteine-binding covalent analogs and covalently docking them to the target protein using DOCKovalent.^55^ Retrospective benchmarking of Covalentizer on a set of covalent kinase inhibitors has shown that it is able to rediscover a substantial portion of the known binders, with a success rate of 40% in identifying the binding mode validated by protein X-ray crystallography. However, its performance is limited by the performance of the covalent docking program, and it allows only marginal receptor flexibility, which can be essential for targeting more flexible side-chains such as lysine.

Here, we present two computational approaches that utilize known binders to design lysine-targeting covalent analogs. We compare two different modeling approaches that address the design problem from the two different sides of the interface: a “ligand-side” approach, adopted from the Covalentizer protocol, in which we install different electrophiles on different ligand vectors and dock each candidate into the binding pocket; and a “protein-side” approach, in which we install the electrophile on the target nucleophilic residue and compare the conformational space of the modified residue to the crystal structure of the protein bound to the noncovalent ligand, to identify possible installation vectors.

We tested these protocols retrospectively on a data set of lysine-binding covalent ligands, as well as on a data set of structures of vitamin B6 bound to different receptor lysines. Our ligand-side protocol was able to recapitulate the correct binding conformation in 80% and 86% of the cases, and our protein-side protocol in 56% and 82% of the cases, respectively. We then applied these protocols prospectively to a data set of known kinase inhibitors and identified thousands of potential covalent candidates for more than 200 human kinases. Two of these candidates have been experimentally validated as new MKK7 inhibitors with IC_50_ values of 738 nM and 695 nM, and covalent binding to the target lysine was confirmed by mass spectrometry and X-ray crystallography.

## Results

### Ligand-Side Protocol

Our ligand-side protocol (Figure 1) follows similar steps to those described in the Covalentizer protocol,^59^ adapted into the Rosetta framework.^60^ Starting from a crystal structure of the target protein with a known noncovalent ligand that binds near a lysine residue (Figure 1A), we install different electrophiles on different ligand positions to generate a library of potential electrophilic analogs (Figure 1B). We use RDKit (RDKit: Open-source cheminformatics. https://www.rdkit.org) to generate a conformer library for each of these analogs, including conformers which are constrained to match the crystallographic conformation of the initial template ligand, as well as unconstrained conformers. Each analog is then introduced to Rosetta, modeled in its adduct form, and docked into the binding pocket using the RosettaLigand high-resolution docking protocol,^61,62^ while applying covalent constraints to enforce the formation of the covalent bond to the target lysine.^63^ The constraints are harmonic functions that favor the placement of the atoms forming the covalent bond in the ideal covalent bond geometry; such placement would result in a constraint score of 0. Candidates that produce a top-10 scoring model with low constraint score (<2) and low RMSD (<1.5 Å) to the crystallographic ligand, which indicates that the candidate is able to fit the covalent bond geometry while maintaining the native noncovalent interactions, are considered high-confidence candidates (Figure 1C).

**Figure 1:**
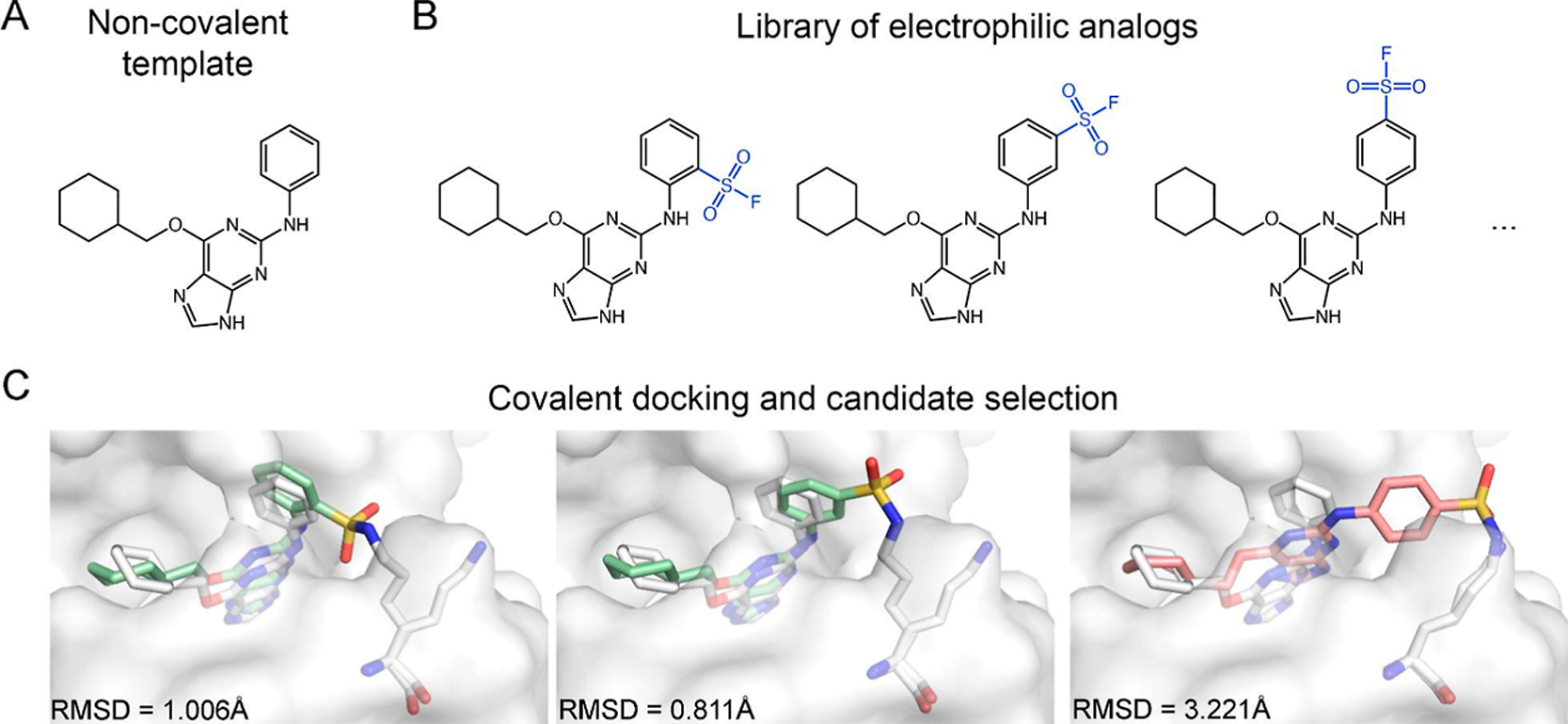
Outline of the ligand-side protocol. (A) We start from a known noncovalent ligand that binds near a receptor lysine residue. (B) We generate a library of electrophilic analogs by installing different warheads on different ligand positions. (C) We dock each analog into the protein while applying covalent constraints to enforce covalent bond formation to the target lysine, and we select high-confidence analogs (green) that yielded top-10 scoring models with constraint score <2 and RMSD < 1.5 Å to the crystallographic conformation (white). Other analogs (pink) are discarded.

To assess the ability of this protocol to identify known covalent binders, we collected 98 complex structures of 60 lysine-binding ligands from the CovPDB database^64^ (Data set S1). To simulate the case of starting from a noncovalent ligand, we removed the electrophilic warhead from each of these ligands, and applied our protocol to each of these “non-covalent” complex structures. The correct binding conformation (constraint score <2 and ligand RMSD < 1.5 Å) was sampled in 90% of the cases and ranked among the 10 top-scoring models in 80% of the cases (Figure 2A). We next tested our protocol on a larger data set of 309 structures of proteins that are covalently bound to a vitamin B6 molecule, collected from the Protein Data Bank (Data set S2). Vitamin B6 contains an isonicotinaldehyde moiety, where the aldehyde can form a reversible covalent imine bond with a lysine amine.^65^ In this data set, the correct binding conformation was sampled in 94% of the cases and ranked among the 10 top-scoring models in 86% of the cases (Figure 2A,C).

**Figure 2:**
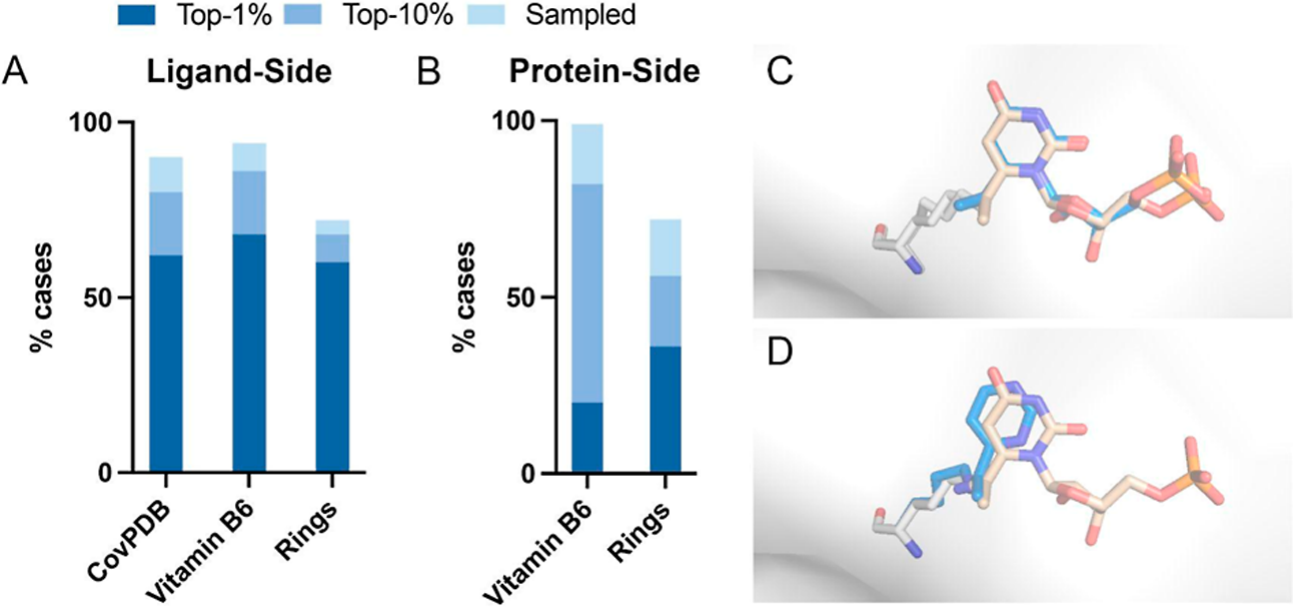
Success rate of modeling lysine covalent adducts. (A) Percent of starting structures for which our ligand-side protocol sampled the correct binding conformation (light blue), ranked it among the ten top-scoring models (blue) or ranked it as the top-scoring model (dark blue), shown for each data set. Similarly, (B) shows the percent of starting structures for which our protein-side protocol sampled the correct ring conformation, ranked it among the 10% top-scoring conformers or ranked it among the 1% top-scoring conformers. (C) Native conformation (beige) and a top-10 scoring model (blue) produced by our ligand-side protocol, with ligand RMSD = 1.5 Å, of PDB ID 3EWU. Similarly, (D) shows a top-10% scoring model produced by our protein-side protocol, with ring RMSD = 1.0 Å, of the same structure.

### Protein-Side Approach

Our protein-side protocol presents a more compact modeling approach, where instead of docking each electrophilic analog as a whole, we model only a small part of the ligand scaffold, onto which we install the electrophile. Starting from a protein–ligand complex structure, we remove the ligand from the structure, and we modify the target lysine with an electrophile installed on a ligand ring moiety, such as a phenyl ring, omitting the rest of the ligand. We then use Rosetta to enumerate all the possible rotamers of this modified residue and to model each rotamer in the context of the binding pocket. For each top-scoring rotamer, we calculate the RMSD between the ring of the modeled modified residue and each similar ring in the crystal structure of the noncovalent ligand. If two rings overlap with RMSD < 1 Å, we reason that installing the electrophile on the appropriate ring position is likely to result in a covalent binder (Figure 3).

**Figure 3:**
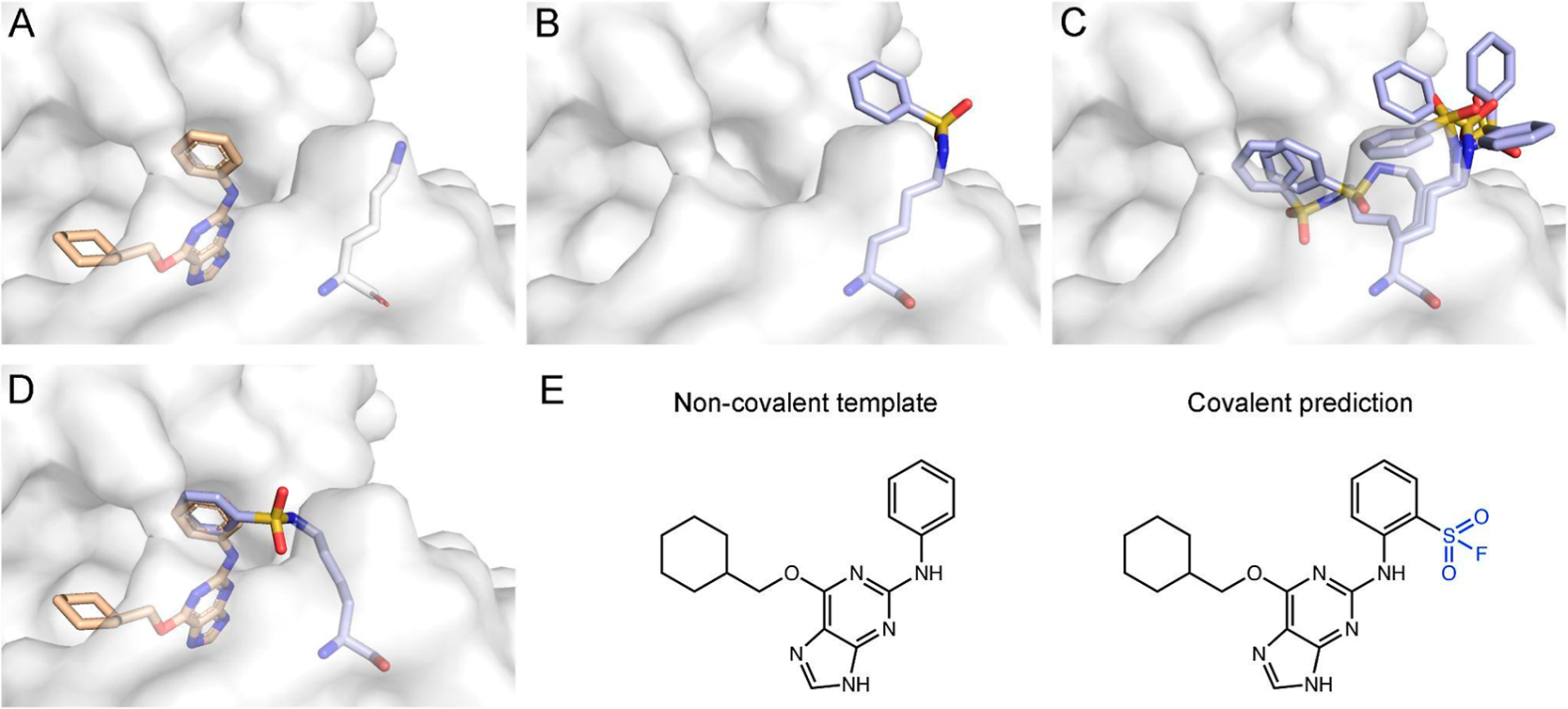
Outline of our protein-side, covalent small-molecules design protocol. (A) We start from a crystal structure of a noncovalent ligand that binds near a receptor lysine. (B) The ligand is removed from the structure and the target lysine is modified with an electrophile installed on a ring moiety, such as an aryl-sulfonyl fluoride. (C) We model the possible rotamers of the modified lysine in the context of the binding pocket. (D) We compare the top-scoring rotamers to the crystallographic conformation to identify models with ring RMSD < 1 Å. (E) High-confidence covalent analogs are suggested accordingly.

To assess the performance of this protocol and compare it to the performance of our ligand-side protocol, we collected a subset of 25 complex structures from our CovPDB data set, in which the electrophile is installed directly on an aromatic ring moiety (Data set S1), and applied our protocol to each of these structures. The correct ring conformation and warhead position (ring RMSD < 1 Å) were sampled in 72% of the cases and ranked among the 10% top-scoring models in 56% of the cases (Figure 2B). In the same subset, our ligand-side protocol sampled the correct binding conformation in 72% of the cases, and ranked it among the 10 top-scoring models in 68% of the cases (Figure 2A). In our vitamin B6 data set, the correct warhead position and ring conformation were sampled in 99% of the cases and ranked among the 10% top-scoring models in 82% of the cases (Figure 2B, left and Figure 2D).

### Design of Lysine-Binding Kinase Inhibitors

Encouraged by the success rate of our protocols in recapitulating known covalent binders, we aimed to use these protocols for the prospective design of new lysine-targeting covalent binders based on known kinase inhibitors. We searched the Protein Data Bank for structures of human kinases bound to a small-molecule that contains a phenyl ring, which has at least one ring atom within 8 Å of a lysine amine. We clustered the results by receptor sequence similarity and ligand fingerprint similarity, resulting in 2132 structures (Data set S3). We then applied our protocols to each of these structures, using two different lysine-targeting electrophiles: a phenyl sulfonyl fluoride warhead^17^ and a 2-hydroxybenzaldehyde warhead.^66^

Our ligand-side protocol produced top-10 scoring models with constraint score <2 and ligand RMSD < 1.5 Å for 7369 different complexes, comprising 6036 electrophilic compounds and 265 kinases. Our protein-side protocol produced top-10% scoring models with ring RMSD < 1 Å for 3301 different complexes, comprising 2787 electrophilic compounds and 224 kinases. There was an overlap of 1620 covalent complexes that were predicted by both protocols.

### Identifying New Lysine-Targeting MKK7 Binders

Of the list of kinases with available predictions, we focused on MKK7, a kinase for which we have previously developed covalent inhibitors.^58,67,68^ A potential candidate that was identified by both protocols is a lysine-targeting analog (compound **1**, Figure 4A) of MKK7-COV-3, a previously discovered cysteine-targeting covalent inhibitor of MKK7 (PDB ID: 5Z1D).^58^ Our results suggested that installing an aldehyde at the para position of the phenyl ring and replacing the acrylamide warhead with a hydroxyl group will allow the ligand to bind Lys221 of MKK7 covalently (Figure 4B, ligand RMSD = 1.3 Å and ring RMSD = 0.7 Å, respectively). To validate this prediction, we synthesized **1**, incubated it with MKK7 (Overnight, 4 °C) and measured its binding via intact protein LC/MS. As the aldehyde warhead is expected to form a reversible covalent imine bond with the lysine amine, the mixture was reduced with NaBH_4_ (5 mM; 1 h; Room temp.) to trap the covalent complex prior to LC/MS measurements. Significant labeling was detected only after the reduction of the complex (Figure 4C), which correlates with the reversible nature of the imine bond.

**Figure 4:**
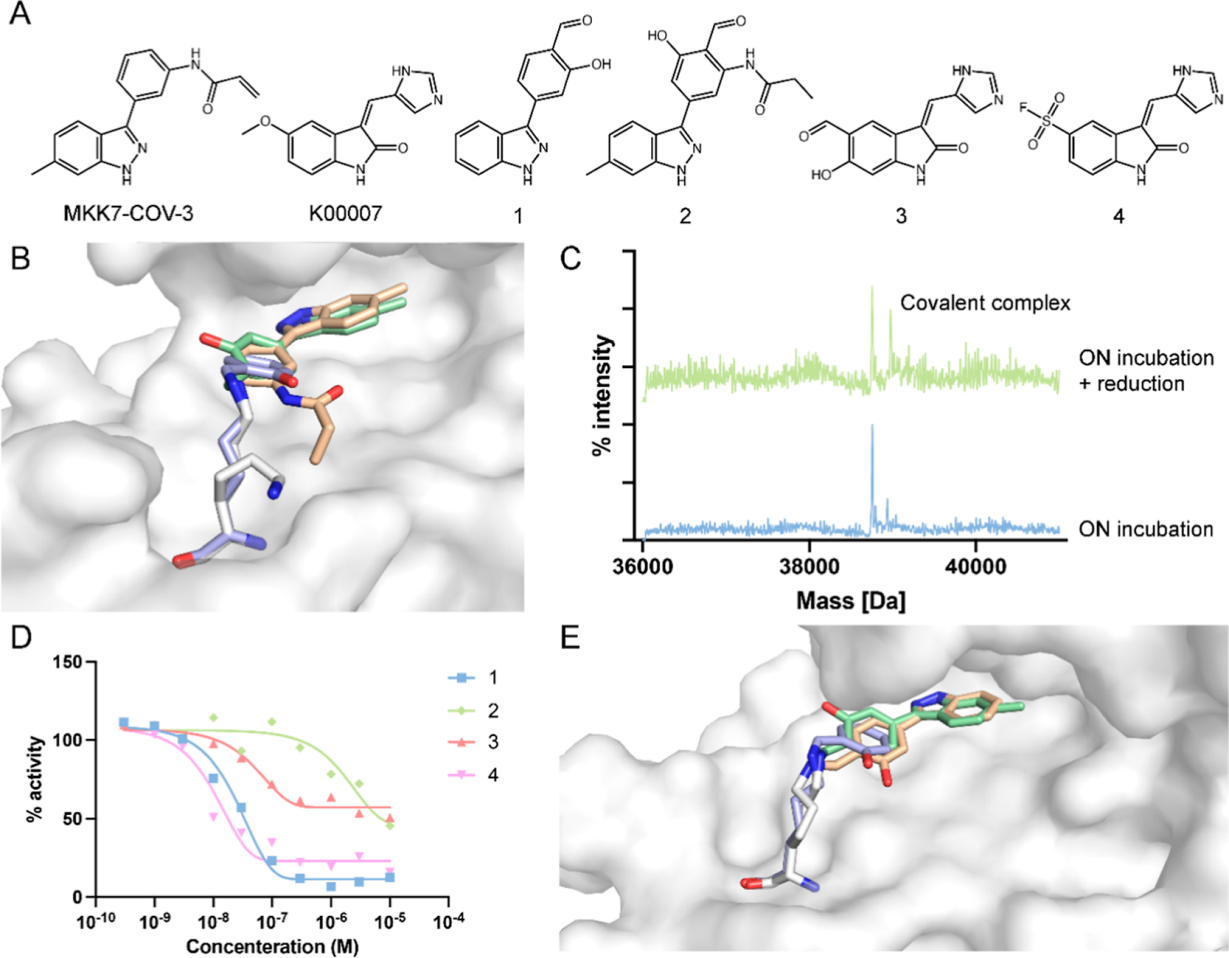
Our protocols successfully identify a new lysine-targeting MKK7 binder. (A) Chemical structures of reported MKK7 inhibitors MKK-COV-3 and K00007 and their derivatized lysine targeting covalent candidates. (B) The modeling prediction of our ligand-side protocol (green) and our protein-side protocol (blue) overlaid on the template binder (beige, PDB ID: 5Z1D). (C) LC/MS spectra of compound **1** after overnight incubation at 4 °C with the protein, and after 1 h reduction with sodium borohydride at room temperature. (D) Inhibition of MKK7 by compounds **1–4** in a kinase activity assay. (E) Co-crystal structure of MKK7 with compound **1** (beige), overlaid on the closest modeling prediction within the top-scoring models of our ligand-side protocol (green) and our protein-side protocol (blue).

Following this result, we prepared three additional compounds: compound **2**, which is based on a second analog of MKK7-COV-3 that was suggested by both of our protocols, where an aldehyde warhead is placed next to the original acrylamide warhead, with the acrylamide reduced to ensure any covalent binding would occur only through the aldehyde warhead; compound **3**, which is an aldehyde analog of the previously discovered noncovalent binder K00007 ^69^ (PDB ID: 6YG4) that was suggested by our ligand-side protocol; and compound **4**, which is a sulfonyl fluoride analog of the same ligand that was suggested by both of our protocols. We evaluated these compounds, as well as control inhibitor Staurosporine, in a kinase activity assay against MKK7. Compounds **1** and **4** showed IC_50_ values of 738 nM and 695 nM, respectively, compared to 1.1 μM for Staurosporine. Compounds **2** and **3** did not show inhibition under these conditions (IC_50_ > 10 μM; Data set S4).

To further confirm the binding site of our compounds, we cocrystallized MKK7 with compound **1** (PDB: 9HZ0; Figures 4E and S1 and Table S1). The crystal structure confirmed Lys221 as the binding site, despite poor electron density at the covalent linkage, it showed a large movement of the lysine side-chain, which is pointing away from the ligand in the original cysteine-bound structure, indicating the formation of the covalent bond. Cys218 remained unmodified. Elevated b-factors and the electron density map indicate flexibility and/or lower occupancy of the ligand (Table S1, Figure S1), additionally this partial occupancy might be the result of the reversible nature of the imine covalent bond, and reflect partial hydrolysis of the bond. Comparison of this structure with the predictions from both of our protocols showed close agreement to the obtained structure, with some minor differences, such as our ligand-side protocol placing the hydroxyl group at the opposite side of the ring, resulting in a slightly higher RMSD value of 1.8 Å (0.5 Å without the hydroxyl), and our protein-side protocol showing a slight rotation of the phenyl ring (Figure 4E). However, no experimental density was observed for the hydroxyl (Figure S1B), hence our prediction might still be accurate.

## Discussion

Covalent chemical probes and drugs can have many advantages over noncovalent compounds. However, their discovery is challenging. In this study, we developed two computational methods to design lysine-targeting covalent binders by installing an electrophile on an already known binder, an approach that proved to be successful in previous experimental and computational studies, and have led to the discovery of many covalent binders. These two methods present two different modeling strategies, with each method addressing the design problem from a different side of the interface: a “ligand-side” approach and a “protein-side” approach.

Our ligand-side protocol uses a similar approach to that of the previously reported Covalentizer protocol, implemented within the Rosetta framework. However, our implementation can offer a few advantages. First, using the RosettaLigand docking protocol, instead of DOCKovalent, adds receptor flexibility to our method, which may allow it to resolve small clashes introduced by the addition of the electrophilic warhead and to yield more accurate predictions. It also allows sampling of different rotamers of the target nucleophilic residue on-the-fly, which may become crucial when targeting a highly flexible residue such as lysine. Second, by generating ligand conformers which are constrained to the crystallographic conformation of the initial template ligand, we can guide the modeling process toward the presumed conformation of the covalent analog, which can significantly improve its performance. Previous benchmarking of RosettaLigand against the Astex Diverse Set of 85 noncovalent protein–ligand complexes^70^ showed that when given a conformer library that includes the crystallographic conformation of the ligand, RosettaLigand is able to produce a top-scoring model with RMSD < 2 Å in 99% of the cases, compared to 58% when the crystallographic conformation is excluded from the library.^61^

Our protein-side protocol presents a more compact modeling approach, where instead of docking the same ligand multiple times with only a slight modification for each candidate, we model only the modified part. Such an approach may reduce the run-time of the protocol, especially for large and flexible ligands that contain multiple instances of the same aromatic rings. For example, to derivatize our kinase inhibitors data set, our ligand-side protocol took an average of approximately 28 single-CPU hours for each structure, with an average of 21 analogs per structure and 82 min to model each analog (12 min for conformer generation and 42 s for each of the 100 docking simulations). On the same data set, our protein-side protocol took an average of approximately 4 single-CPU hours for each structure, with an average of 2411 rotamers for each of the two warheads and 3 s to model each rotamer (we note that since Rosetta uses backbone-dependent rotamer libraries, the number of rotamers can vary between structures).

On the other hand, our protein-side approach has a few limitations. First, it requires the ligand to have a rigid structural moiety, such as an aromatic ring, in the vicinity of the target lysine (Figure 2D). While aromatic rings are very prevalent in drug design, many natural and synthetic ligands do not have a suitable ring moiety in close proximity to a receptor lysine, and therefore cannot be derivatized using our protein-side protocol. Second, since the modeling is performed with only a small part of the ligand in the binding pocket, it may generate conformations that cannot be adopted when the entire ligand is present. For example, the electrophile may clash with another ring substituent, or a neighboring side-chain may be shifted too close to a different part of the ligand. Some of these cases may be resolved by simply removing the clashing substituent, however in other cases, a different modeling approach, such as our ligand-side protocol, may be required to properly model the covalent analog. In practice, the slightly better retrospective performance and broader applicability (beyond aryl-electrophiles) of the ligand-side approach make it the preferred choice. However, when possible, we recommend using both approaches and prioritizing hits that are identified by both protocols. The fact that compounds **2** and **3** failed to potently inhibit MKK7 despite using the same electrophile as **1**, suggests there is also room for improvement in the accuracy of the modeling, since apparently the true positioning of these ligands did not elicit efficient covalent bond formation.

Despite their limitations, we were able to successfully apply these protocols to transform two known MKK7 inhibitors, one noncovalent and one cysteine-binding, into two new lysine-binding MKK7 inhibitors. Compound **1** was confirmed to covalently bind to Lys221 using X-ray crystallography. While the original ligand in this case also exhibits covalent binding to the protein, binding to a different nucleophile may result in different kinetics and selectivity profile, which may be more suitable for different applications.

Searching through the entire PDB, we identified 57,895 lysine residues that are located near a known small-molecule binder in 20,291 different proteins, suggesting that many of these proteins may be amenable to covalent targeting through our ligand-side protocol. 17,941 lysines in 9227 different proteins are located near an aromatic ligand atom, demonstrating the more limited yet significant scope of our protein-side protocol. We should note that while in this work we only explored two electrophiles, the approach is modular and several additional electrophiles demonstrated to be able to modify lysines can be implemented, further expanding its scope. Overall, we believe these methods can largely facilitate the discovery of new covalent probes for a wide range of targets and applications.

## Methods

### Generating Electrophilic Analogs

To generate a library of electrophilic analogs of a template ligand, we use the RDKit Chem.rdChemReactions module and SMARTS-based language. The allowed reactions include the installation of an aldehyde and a hydroxyl group on two adjacent phenyl ring atoms, where the aldehyde can react to form a Schiff base with a lysine amine;^66^ and the installation of a sulfonyl fluoride on a phenyl ring, where the sulfonyl fluoride can undergo a SuFEx reaction.^17^ Existing substituents on the reacting positions are removed. Reactions that break a ring bond are excluded. For benchmarking, we included the reactions represented in the benchmarking data set. The analogs are generated in their reacted form, and include the lysine Nζ and Cε atoms.

### Parametrizing Covalent Residues for the Ligand-Side Protocol

For each reacted covalent analog, we use RDKit AllChem.EmbedMolecule function to generate up to 100 unconstrained conformers and up to 100 conformers where the maximum common substructure, calculated using the Chem.rdFMCS module with the BondCompare = CompareAny option, is constrained to fit the crystallographic conformation of the template ligand. Each conformer is minimized using UFF force field calculations with the Chem.rdForceFieldHelpers module. To verify that the resulting conformation is chemically valid, we calculate the energy of the conformer, and discard conformers with energy that exceeds the energy of the template ligand by more than 100 units. Each conformer is then aligned to the crystallographic conformation of the template ligand based on their maximum common substructure. We keep only conformers with RMSD > 0.5 Å to all previous conformers. Lastly, we delete the lysine Cε atom, and replace the lysine Nζ atom with a vanadium atom, which is interpreted by Rosetta as a virtual atom.

To parametrize the ligand for use in Rosetta as a covalently linked residue, we first use the molfile_to_params.py script provided in the Rosetta software suite to create a ligand params file. To indicate that the ligand is covalently bound to another residue, we add a CONNECT record to the residue params file, specifying which atom participates in the inter-residue covalent bond, as described in Drew et al.^71^ The connection is given the same internal coordinates as the virtual atom, which represents the atom at the other end of the covalent bond.

Similarly, we create a suitable covalently linked variant of lysine for each electrophile. We implement these variants through the residue patch system, to utilize the existing definitions and rotamer libraries that have been optimized for use in Rosetta.^72^ We first use the GaussView interface and the Gaussian software optimization procedure, as described in Renfrew et al.,^73^ to draw and optimize a N-acetylated, C-amidated lysine, with the side-chain amine modified with an atom of the same type as the ligand atom at the other end of the covalent bond. We use the OpenBabel toolbox (http://openbabel.org) to convert the optimized structure into a mol file, and then to a Rosetta params file using the molfile_to_params_polymer.py script provided in the Rosetta software suite. We then create a patch file that adds a virtual atom and a CONNECT record to allow covalent binding, updates the number of hydrogens, charges and atom types as necessary, and adds a PROTON_CHI record to allow sampling of the new rotamers around the bond CE-NZ bond that are added due to the covalent binding.

### Covalent Ligand Docking

We dock each electrophilic analog into the binding pocket using the RosettaScripts interface.^74^ Starting from the input protein–ligand complex template structure, we first identify the chains that have at least one atom within 10 Å from any ligand atom, and remove all other chains, as well as the template ligand, using the clean_pdb.py script provided in Rosetta. We prepack the structure using the PackRotamersMover and MinMover protocols. We then add the electrophilic analog into the structure, and use the high-resolution modeling steps and score functions used in the ligand docking XML protocol^62^ to generate 100 models of the covalent complex, while applying AtomPair constraints between each of the covalent bond actual atoms and its virtual placeholder in the partnering residue. We use the HARMONIC score function, centered at 0 and with a standard deviation of 0.3.

Models are ranked by their interface score. For each model, we calculate the RMSD between the modeled conformation of the ligand and the crystallographic conformation of the template ligand using RDKit, based on their maximum common substructure, computed using the Chem.rdFMCS module with the BondCompare = CompareAny option.

### Parametrizing Modified Lysines for the Protein-Side Protocol

To parametrize our modified lysine residues for use in Rosetta, we follow the steps described in Renfrew et al.^73^ We use the GaussView interface to draw a N-acetylated, C-amidated lysine, which we modify with the electrophile installed on a ring moiety. We then use the Gaussian software to optimize the structure. We convert the optimized structure to a mol file using the OpenBabel toolbox (http://openbabel.org), and then to a Rosetta params file using the molfile_to_params_polymer.py script provided in the Rosetta software suite. As Rosetta allows only four CHI records to define the side-chain dihedrals, we use PROTON_CHI records to define the additional rotatable bonds. Rotamer libraries are generated using the Rosetta MakeRotLib application.

### Modeling the Conformational Space of the Modified Lysine

To model the modified lysine residue in the context of the binding pocket, we use the RosettaScripts interface.^74^ Starting from the input protein–ligand complex template structure, we first identify the chains that have at least one atom within 10 Å from any ligand atom, and remove all other chains using the clean_pdb.py script provided in Rosetta. We add the template ligand back into the structure and minimize the protein around the ligand using the MinMover protocols with tolerance = 0.00001. We then remove the ligand from the structure. We use the MutateResidue mover to modify the target lysine with the electrophile, and the TryRotamers mover to sample each rotamer from the pregenerated rotamer library. To sample all possible rotamers, we recompiled Rosetta and changed the while-loop condition in the fill_rotamer_vector function in the/core/pack/dunbrack/RotamericSingleResidueDunbrackLibrary.tmpl.hh file to “count_rotamers_built < max_rots_that_can_be_built”, and we break the loop if we reach a rotamer with zero probability. To reduce execution time, we set the extra rotamers sampling in the setup_rotamer_set function in the/protocols/protein_interface_design/movers/TryRotamers.cc file to “false”. For each sampled rotamer we then use the Neighborhood residue selector and the MinMover with tolerance = 0.00001 to minimize the side-chains of all residues within 10 Å from the modified lysine. Finally, we use the TotalEnergyMetric to calculate the score of the 10 Å neighborhood of the modified lysine.

Models are ranked by the score of the 10 Å neighborhood of the modified lysine. For each model, we calculate the RMSD between the ring moiety of the modified lysine and each similar ring in the crystallographic conformation of the initial template ligand, taking ring symmetry into account.

### Benchmarking Data Sets

To construct our CovPDB data set, we searched the CovPDB database^64^ for structures that include a lysine-binding ligand, which yielded 204 structures. We discarded 65 ligands that react through a nonterminal or substituted warhead. We excluded 41 additional structures due to different structure-related issues, such as a missing LINK record, a ligand structure that does not match the SMILES string on the CovPDB database, or an irregular covalent bond length (e.g., a distance of 1.95 Å between the P atom of AMP and the NZ atom of Lys25 in PDB ID 4D05, which is fragmented by RDKit). This yielded 98 structures which were used to test our ligand-side protocol. We then filtered these structures for ligands in which the electrophilic warhead is installed directly on an aromatic ring moiety, resulting in a subset of 25 structures, which were used to test our protein-side protocol.

To construct our vitamin B6 data set, we downloaded from the PLP ligand page on the PDB Web site a list of 1063 structures in which the ligand is present as a nonpolymer residue, covalently linked to another residue. We filtered this list for structures in which the PLP ligand has a single LINK record that connects its C4A atom to a protein lysine NZ atom. We then clustered the results using the PDB sequence clusters with 90% sequence identity, keeping only one structure of each receptor lysine. This yielded 324 structures. Fifteen structures were excluded due to different structure-related issues, such as a covalent linkage to a nonpolymer lysine (e.g., PDB ID 1KO0) or an irregular bond length (e.g., a distance of 1.94 Å between the C4A atom of PLP and the NZ atom of Lys276 in PDB ID 2HZP, which is fragmented by RDKit), resulting in 309 structures.

### Kinase Binders Data Set

We collected a list of UniProt accession codes of human kinases from the UniProt knowledgebase (https://ftp.uniprot.org/pub/databases/uniprot/current_release/knowledgebase/complete/docs/pkinfam.txt). We then searched the PDB using the following attributes: (1) the experimental method is X-ray diffraction, (2) the structure contains any of the accession codes in the UniProt list, and (3) it contains a ligand that include a phenyl ring as a substructure. This search yielded 6113 structures. We searched these structures for ligands that have at least one phenyl ring atom within 8 Å from a lysine NZ atom. To verify that the lysine belongs to a human kinase chain, we compared the UniProt accession code listed in the DBREF records in the PDB file to our UniProt list. We clustered the results with respect to both the receptor, using the PDB sequence clusters with 90% sequence identity, and the ligand, using RDKit fingerprint similarity calculation with a 0.6 similarity cutoff, using the Chem.RDKFingerprint and DataStructs.FingerprintSimilarity functions. This yielded 2132 structures of 288 kinases bound to 1831 ligands.

### PDB-wide Search

We searched the PDB using the following attributes: (1) the experimental method is either X-ray diffraction or electron microscopy, (2) the structure contains at least one protein chain and (3) the structure contains at least one nonpolymer chain. This search yielded 169,656 structures. We searched these structures for ligands that have at least one atom within 8 Å from a lysine NZ atom, excluding cases where the ligand has a LINK record connecting the ligand to the same NZ atom, indicating they are already covalently bound. We clustered the results with respect to the receptor lysine, using the PDB sequence clusters with 90% sequence identity and the lysine sequence position, and the ligand PDB 3-letters identifier. We filtered out ligands containing less than 10 atoms, to avoid irrelevant molecules such as ions and solvents. This yielded 112,005 unique lysine–ligand pairs, consisting of 23,403 different ligands and 57,895 lysine residues in 20,291 different proteins. 42,556 of these pairs have an aromatic ligand atom near the lysine, making them susceptible to covalent targeting through our protein-side protocol, including 16,620 ligands and 17,941 lysine in 9227 proteins.

### MKK7 Ligand Selection

Using the ligand-side protocol 48 candidates met the selection criteria (constraint score <2 and RMSD < 1.5). With the protein-side protocol 12 MKK7 candidates passed the criteria of top 10% neighborhood score and ring RMSD < 1. We visualized the predictions and selected compounds based on synthetic feasibility and diversity (i.e., we chose compounds from two disparate scaffolds). All compounds that were synthesized and tested are reported in the paper.

### Binding Experiments of MKK7 Binder

His-tagged MKK7 kinase domain was diluted to 2 μM in HEPES 20 mM pH = 7.5, 150 mM NaCl. The protein was incubated overnight at 4 °C with different concentrations of the aldehyde-containing molecule. Following the incubation, samples were either directly injected to LC/MS, or incubated with 5 mM freshly dissolved sodium borohydride for 1 h at room temperature, followed by injection to LC/MS.

### MKK7 Kinase Activity Assays

MKK7 kinase activity assays were performed by Reaction Biology. The reaction was monitored using a radiometric protein kinase assay (^33^PanQinase Activity Assay), using GST or His-tagged kinase in ScintiPlates from PerkinElmer. The kinase and substrate were preincubated with compound at room temperature for 30 min, followed by addition of ATP and incubation at 30 °C for 60 min. The buffer contained 70 mM HEPES-NaOH pH 7.5, 3 mM MgCl_2_, 3 mM MnCl_2_, 3 μM Na-orthovanadate, 1.2 mM DTT, 50 μg/mL PEG20000, 3 μM ATP, [γ-^33^P]-ATP (approximately 8 × 10°^56^ cpm per well), 75.6 nM MKK7 and 2 μg/50 μL JNK1 K55R K56R substrate (kinase dead). The reaction cocktails were incubated at 30 °C for 60 min. The reaction was stopped with 50 μL of 2% (v/v) H_3_PO_4_, plates were aspirated and washed two times with 200 μL 0.9% (w/v) NaCl. Incorporation of ^33^Pi was determined with a microplate scintillation counter (Microbeta, Wallac).

### MKK7 Expression & Purification

For protein crystallization a MKK7 construct containing amino acids 117–423 with a noncleavable N-terminal His6-tag was used. The expression was performed in BL21 DE3 Escherichia coli at 18 °C for 20 h with a subsequent lysis by French press. The nonsoluble components were separated by centrifugation and the supernatant was purified using Ni-affinity chromatography (Qiagen Ni-NTA Superflow 5 mL). After washing with buffer A (50 mM Tris, 500 mM NaCl, 25 mM imidazole, 5% glycerol, 1 mM DTT, pH 8) MKK7 was eluted with a gradient of buffer B (50 mM Tris, 500 mM NaCl, 500 mM imidazole, 5% glycerol, 1 mM DTT, pH 8). The protein was concentrated and further purified by gel filtration chromatography (GE HiLoad 16/600 75 pg) using buffer C (25 mM Tris. 125 mM NaCl, 10% glycerol, 1 mM DTT, pH 7). The eluted protein fractions were combined and concentrated to 30 mg/mL before flash freezing in liquid nitrogen.

### MKK7 Crystallization

The crystallization of apo-MKK7 was performed based on conditions previously described.^68^ Using the hanging drop vapor diffusion method, 1 μL of protein solution (10–15 mg/mL) was mixed with 1 μL of reservoir solution (180–220 mM sodium citrate, 15–25% PEG3350). After 3 days of incubation at 4 °C, prismatic shaped crystals were obtained. Subsequently the apo-crystals were soaked in a separate reservoir solution containing 10% (v/v) glycerol and 5 mM of compound for 24 h, before flash cooling in liquid nitrogen.

### Data Processing and Refinement

Data was collected at the PXII X10SA beamline of the Swiss Light Source (PSI, Villigen, Switzerland) and processed using XDS/XDSAPP (version 3.1.5).^75,76^ Initial phases were obtained by molecular replacement using Phaser^77^ and template 6QFL followed by manual adjustments in Coot^78^ and refinement with phenix.refine.^79^ Inhibitor topology files were generated with phenix.elbow.^79^ Data collection and structure refinement statistics are provided in Table S1. Figures were prepared using The PyMOL Molecular Graphics System.

## Supplementary Material







## Data Availability

All of the protocols described in this work were implemented in Rosetta, which is freely available for academia (https://rosettacommons.org/software/download/). The implementation is described in detail in the methods section. RosettaScripts and example implementations are provided for both protocols as Supporting Information Files. Benchmarks used for validation are available in the Supporting Information as Data sets S1 & S2. The structures of compounds **1–4** and their IC_50_ against MKK7 are available in machine readable form in Data set S4. The crystal structure of MKK7 in complex with compound **1** is deposited to the PDB (9HZ0).

## Notes

Supplementary Figure (S1) and Table (S1) describing X-ray crystallographic analysis of compound 1 bound to MKK7 and crystallographic statistics. Supplementary Chemistry section including synthetic routes, 1H NMR analysis and spectra, LC/MS analysis and spectra of compounds 1–4 (PDF)
List of complexes in our CovPDB data set, and the ‘rings’ subset (Data set S1); List of complexes in our Vitamin B6 data set (Data set S2); List of humen kinase structures used for prospective covalent ligand design (Data set S3); Activities of designed compounds against MKK7 in machine readable format (Data set S4) (XLSX)
Scripts and examples for implementation of the ligand-side and protein-side protocols (ZIP)
