# Supplementary material for: Computational Design of Lysine Targeting Covalent Binders Using Rosetta
Source: J Chem Inf Model. 2025 May 29;65(11):5612–22. doi: 10.1021/acs.jcim.5c00212 (PMC12152945; doi:10.1021/acs.jcim.5c00212)
Supplement: Supplementary file 1 [file ci5c00212_si_001.pdf]

Computational design of lysine targeting covalent binders using Rosetta

Barr Tivon[1], Jan Wiese[2], Matthias Müller[2], Ronen Gabizon[1], Daniel Rauh[2], Nir London[1,#]

[1] Department of Chemical and Structural Biology, The Weizmann Institute of Science, Rehovot 7610001, Israel
[2] Department of Chemistry and Chemical Biology, TU Dortmund University and Drug Discovery Hub Dortmund (DDHD), Zentrum für Integrierte Wirkstoffforschung (ZIW), Otto-Hahn-Strasse 4a, Dortmund 44227, Germany

[#]For correspondence: nir.london@weizmann.ac.il

Supplementary Figure

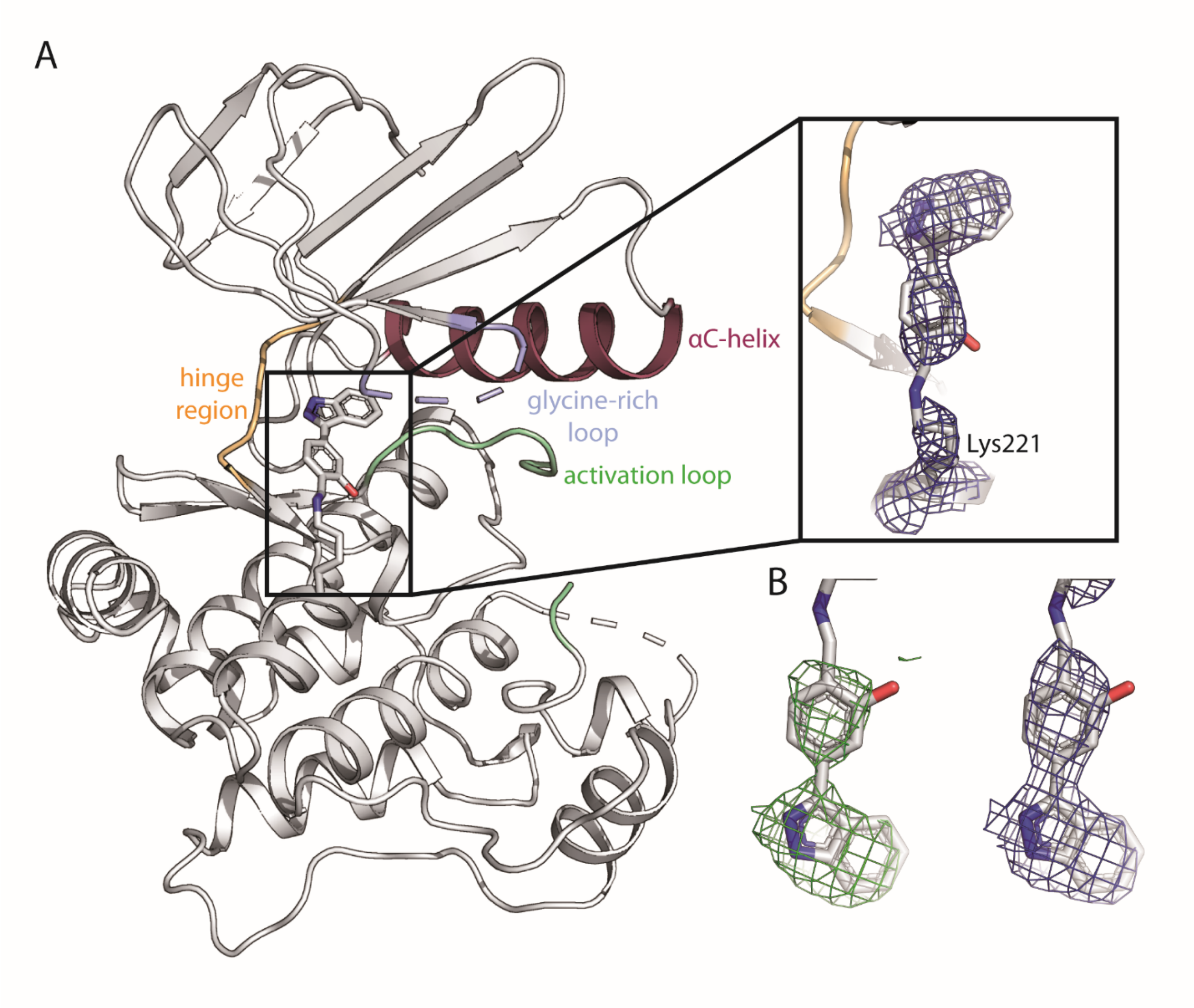

Figure S1. X-Ray crystallographic analysis of compound 1 in complex with MKK7. (A) Overview of the kinase domain with important regions highlighted. The inlet shows compound 1 covalently bound to Lys221 and the corresponding $2mF_O$-DF_C-map contoured at 1.0 r.m.s.d. **(B)** mF_O-DF_C-simulated annealing omit map (left, green, contoured at 2.5 RMSD) and $2mF_O$-DF_C-map (blue, right, contoured at 1.0 RMSD).

Supplementary Table

Table S1. Crystalogaphic statistics

**Data collection**	
PDB ID	9HZ0
Space group	$P\ 2_1\ 2_1\ 2_1$
Wavelength (Å)	1.000020
Cell dimensions<br>a, b, c, (Å)<br>α, β, γ (°)	 61.01 69.14 83.53<br>90 90 90
Resolution (Å)	50.0 - 2.25 (2.35 - 2.25)
R_{meas} (%)	6.9 (255.9)
I/σI	18.58 (1.08)
$CC_{1/2}$	100 (65.8)
Completeness (%)	99.6 (99.3)
Redundancy	13.3 (13.7)
**Refinement**	
Resolution (Å)	45.75 - 2.25
Number of reflections	17231
R_{work} /R_{free} (%)	22.13/26.32
Number of atoms<br>Protein<br>Ligands/ions<br>Water	 2160<br>17<br>18
B-factors<br>Protein<br>Ligands/ions<br>Water	 77.1<br>84.2<br>76.3
Standard deviations<br>Bond lengths (Å)<br>Bond angles (°)	 0.002<br>0.528
Ramachandran plot<br>preferred (%)<br>allowed (%)<br>not allowed (%)	 98.52<br>1.48<br>0

Supplementary Chemistry

Compounds **1-4** were synthesized by WuXi AppTec. We provide their synthetic route and analytical characterization below. The compounds internal names are:

Compound **1**: WEIZ-WX-04-008

Compound **2**: WEIZ-WX-04-011

Compound **3**: WEIZ-WX-04-009

Compound **4**: WEIZ-WX-04-010

<u>WEIZ-WX-04-008</u>

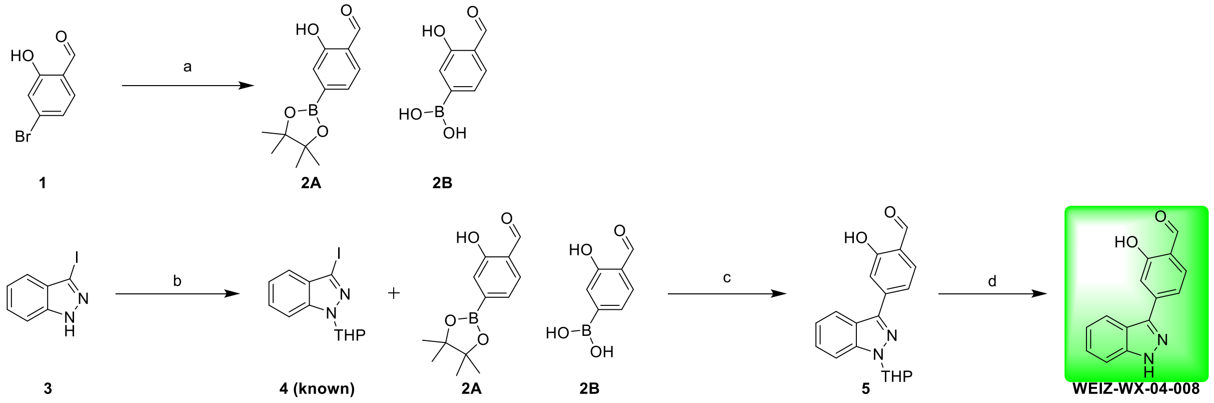

Scheme 1: synthetic scheme: (a) B_2pin_2, $Pd(dppf)Cl_2$, KOAc, dioxane, 80 °C, 4 h (b) DHP, PTSA, EtOAc, 80 °C, 16 h (c) Pd_2dba_3, K_3PO_4, PPh_3, DMF, 80 °C, 4 h (d) TEA, DCM, 25 °C, 2 h.

2-hydroxy-4-(4,4,5,5-tetramethyl-1,3,2-dioxaborolan-2-yl)benzaldehyde:

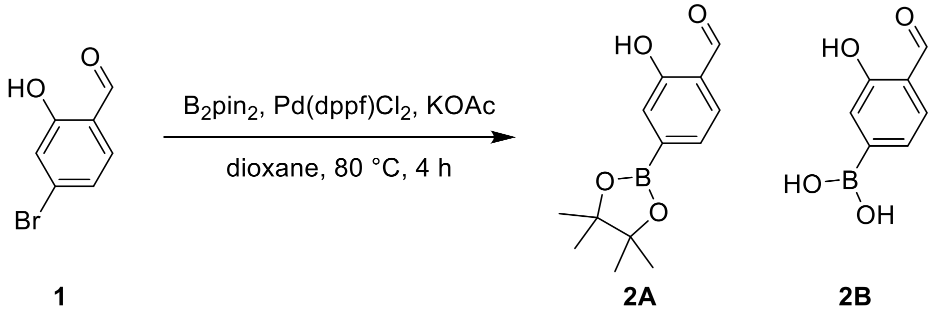

A mixture of 4-bromo-2-hydroxy-benzaldehyde (1 g, 4.97 mmol, 1 eq), 4,4,5,5-tetramethyl-2-(4,4,5,5-tetramethyl-1,3,2-dioxaborolan-2-yl)-1,3,2-dioxaborolane (2.53 g, 9.95 mmol, 2 eq),

KOAc (976.46 mg, 9.95 mmol, 2 eq) in dioxane (10 mL) was degassed and purged with N_2 for 3 times at 20°C, and then the mixture was added Pd(dppf)Cl_2 (364.00 mg, 497.47 umol, 0.1 eq) and stirred at 80°C for 4 h under N_2 atmosphere. The reaction mixture was poured into (100 mL), extracted with Ethyl acetate (80 mL× 3), the combined organic layers were washed with brine (100 mL*2), dried over Na_2SO_4, filtered and concentrated under reduced pressure to give a residue. The residue was purified by column:(SiO_2, Petroleum ether : Ethyl acetate = 5 :1, Rf = 0.5) to give a orange solid (1.2 g, 4.84 mmol, 97.29% yield).

LC-MS (m/z): Calculated: 248.12/166.04; Found: 249.0/166.9[M+H] $^+$.

^{1}H NMR (400 MHz, DMSO) δ: 10.61 (s, 1H), 10.32 (s, 1H), 7.93 (s, 1H), 7.63 (d, J = 7.6 Hz, 1H), 7.30 (s, 1H), 7.20 (d, J = 7.6 Hz, 1H), 1.30 (s, 12H).

3-iodo-1-tetrahydropyran-2-yl-indazole:

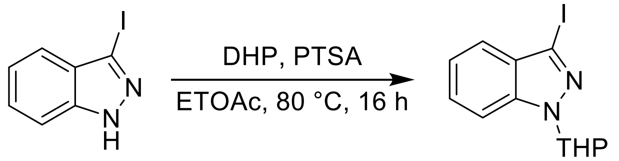

3 **4 (known)**

A mixture of 3-iodo-1H-indazole (1 g, 4.10 mmol, 1 eq) in EtOAc (10 mL) was added 3,4-dihydro-2H-pyran (689.39 mg, 8.20 mmol, 749.33 uL, 2 eq) and PTSA (141.13 mg, 819.57 umol, 0.2 eq) at 20 °C, and then the mixture was stirred at 80 °C for 16 h under N_2 atmosphere. The reaction mixture was purified by column:(SiO_2, Petroleum ether : Ethyl acetate = 5 :1, Rf =0.7) to give a a white solid (1.1 g, 3.35 mmol, 81.80% yield).

LC-MS (m/z): Calculated: 328.01; Found: 329.0 [M+H] $^+$.

^{1}H NMR (400 MHz, DMSO) δ: 7.75 (d, J = 8.4 Hz, 1H), 7.51 (ddd, J = 8.4, 7.2, 1.2 Hz, 1H), 7.45 (d, J = 8.0 Hz, 1H), 7.31-7.22 (m, 1H), 5.86 (dd, J = 9.6, 2.4 Hz, 1H), 3.94-3.82 (m, 1H), 3.78-3.61 (m, 1H), 2.45-2.28 (m, 1H), 2.06-1.91 (m, 2H), 1.82-1.65 (m, 1H), 1.62-1.54 (m, 2H).

2-hydroxy-4-(1-tetrahydropyran-2-ylindazol-3-yl)benzaldehyde:

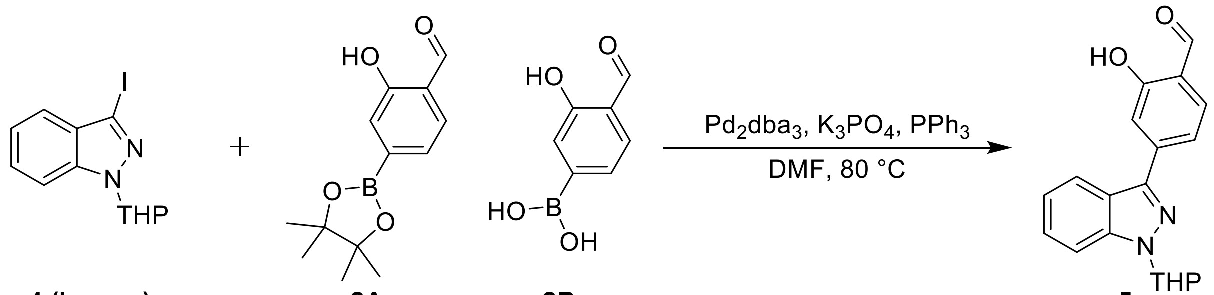

4 (known) **2A** **2B** **5**

A mixture of 3-iodo-1-tetrahydropyran-2-yl-indazole (350 mg, 1.07 mmol, 1 eq), 2-hydroxy-4-(4,4,5,5-tetramethyl-1,3,2-dioxaborolan-2-yl)benzaldehyde (317.52 mg, 1.28 mmol, 1.2 eq) K_3PO_4 (679.20 mg, 3.20 mmol, 3 eq) and tris-o-tolylphosphane (486.95 mg, 1.60 mmol, 1.5 eq) in DMF (3.5 mL) was degassed and purged with N_2 for 3 times at 20 °C, and then the mixture was added $Pd_2(dba)_3$ (97.67 mg, 106.66 umol, 0.1 eq) stirred at 80 °C for 4 h under N_2 atmosphere. The reaction mixture was poured into (40 mL), extracted with Ethyl acetate (30 mL× 3), the combined organic layers were washed with brine (20 mL*2), dried over Na_2SO_4, filtered and concentrated under reduced pressure to give a residue. The residue was purified by column:(SiO_2, Petroleum ether : Ethyl acetate = 1:1 , Rf =0.3) to give a red solid (80 mg, 248.17 umol, 23.27% yield) was obtained as a red solid.

LC-MS (m/z): Calculated: 322.13; Found: 323.0 $[M+H]^+$.

^{1}H NMR (400 MHz, DMSO) δ: 10.86 (s, 1H), 10.33 (s, 1H), 8.11 (d, J = 8.4 Hz, 1H), 7.90-7.75 (m, 2H), 7.69 (d, J = 1.2 Hz, 1H), 7.62 (dd, J = 8.0, 0.8 Hz, 1H), 7.51 (td, J = 7.6, 0.8 Hz, 1H), 7.34 (t, J = 7.6 Hz, 1H), 5.97 (dd, J = 9.6, 2.0 Hz, 1H), 3.91 (d, J = 11.2 Hz, 1H), 3.85-3.72 (m, 1H), 2.48-2.42 (m, 1H), 2.17-2.00 (m, 2H), 1.87-1.73 (m, 1H), 1.69-1.56 (m, 2H).

2-hydroxy-4-(1H-indazol-3-yl)benzaldehyde:

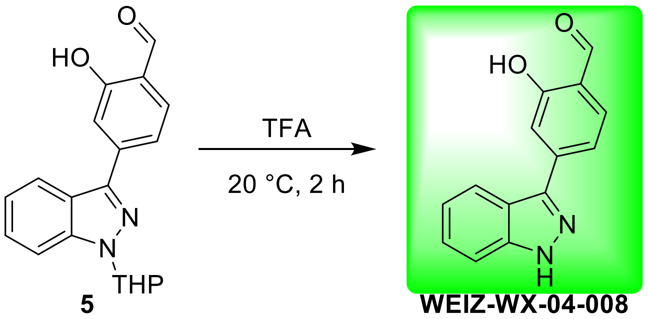

To a solution of 2-hydroxy-4-(1-tetrahydropyran-2-ylindazol-3-yl)benzaldehyde (40 mg, 124.09 umol, 1 eq) in TFA (1 mL). The mixture was stirred at 20 °C for 2 hr. The reaction mixture was concentrated in vacuo. The reaction mixture was purified by prep-HPLC: (FA conditions). The solution was lyophilized to give a yellow solid (9.41 mg, 38.79 umol, 31.26% yield).

LC-MS (m/z): Calculated: 238.07; Found: 238.9$[M+H]^+$. Purity: 98%.

^{1}H NMR (400 MHz, DMSO-d6) δ: 13.50 (s, 1H), 10.84 (s, 1H), 10.31 (s, 1H), 8.10 (d, J = 8.0 Hz, 1H), 7.79 (d, J = 8.0 Hz, 1H), 7.69 (s, 1H), 7.66-7.60 (m, 2H), 7.44 (t, J = 7.6 Hz, 1H), 7.28 (t, J = 7.6 Hz, 1H).

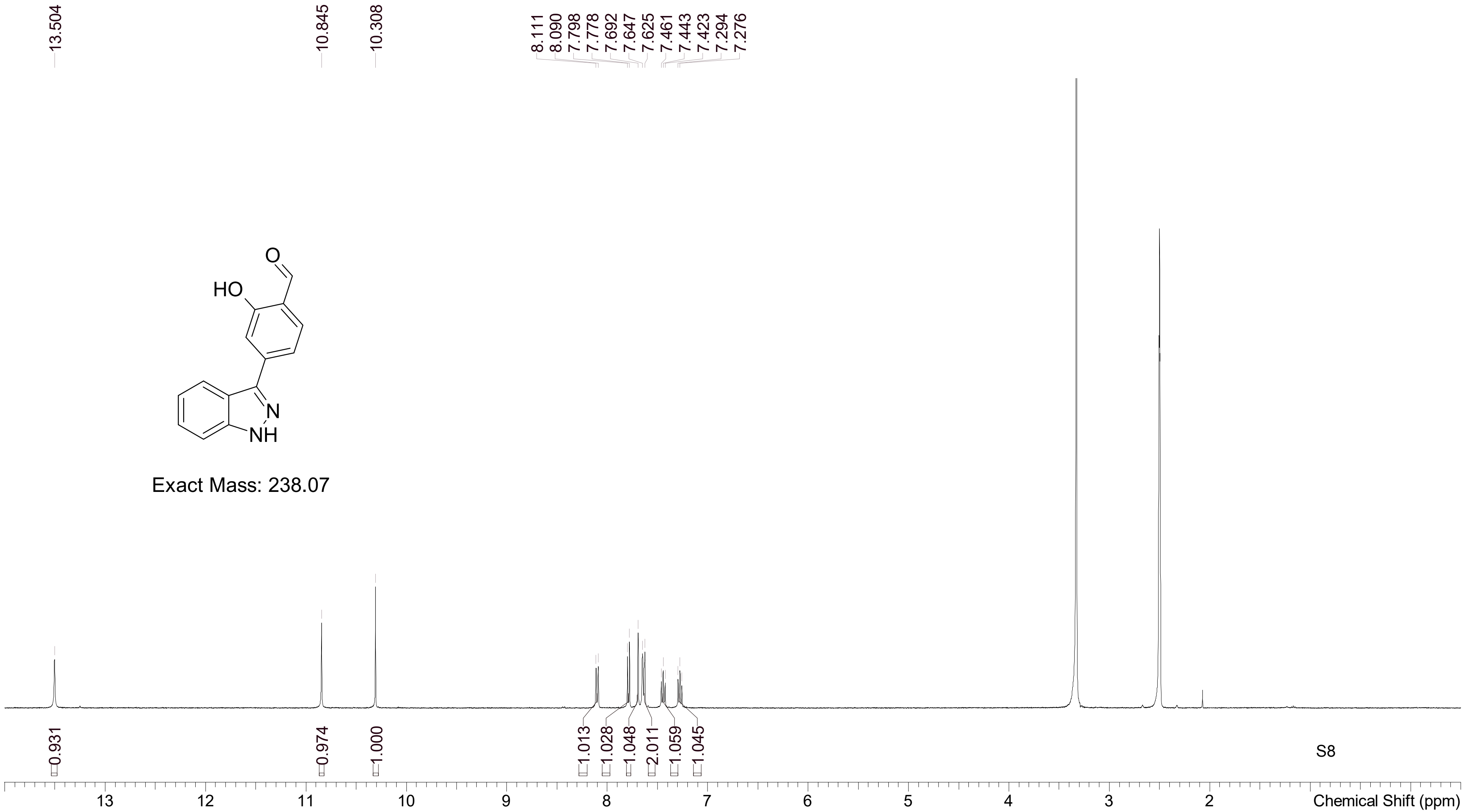
13.504
10.845
10.308
8.111 8.090 7.798 7.778 7.692 7.647 7.625 7.461 7.443 7.423 7.294 7.276
HO
O
N
NH
Exact Mass: 238.07
0.931
0.974
1.000
1.013 1.028 1.048 2.011 1.059 1.045
13 12 11 10 9 8 7 6 5 4 3 2
Chemical Shift (ppm)

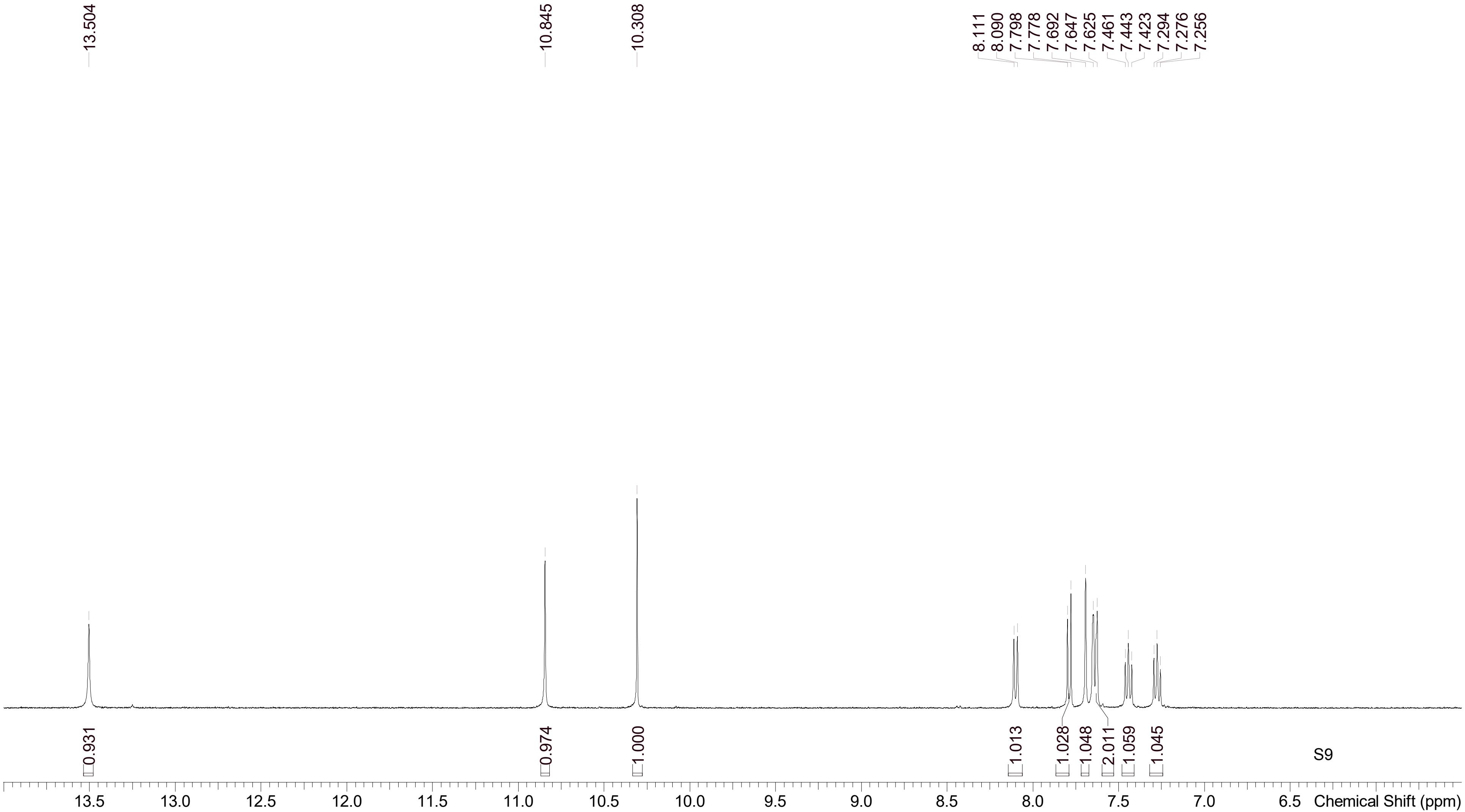
13.504
10.845
10.308
8.111
8.090
7.798
7.778
7.692
7.647
7.625
7.461
7.443
7.423
7.294
7.276
7.256
0.931
0.974
1.000
1.013
1.028
1.048
2.011
1.059
1.045
13.5
13.0
12.5
12.0
11.5
11.0
10.5
10.0
9.5
9.0
8.5
8.0
7.5
7.0
6.5
Chemical Shift (ppm)

S9

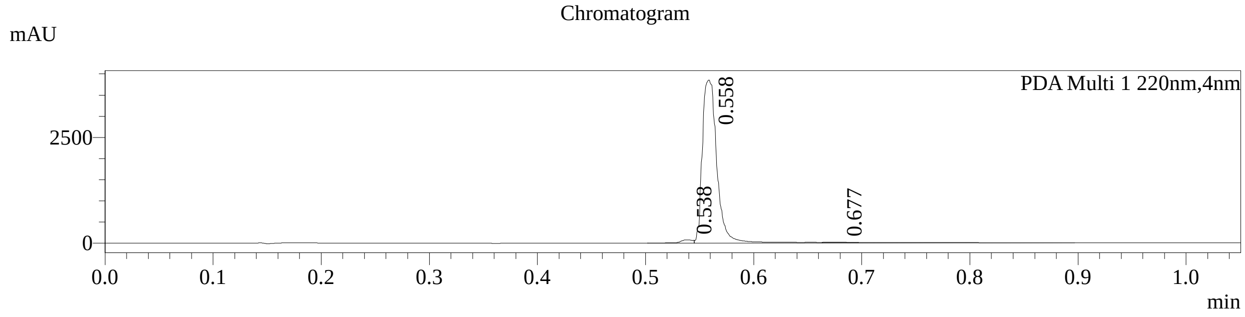

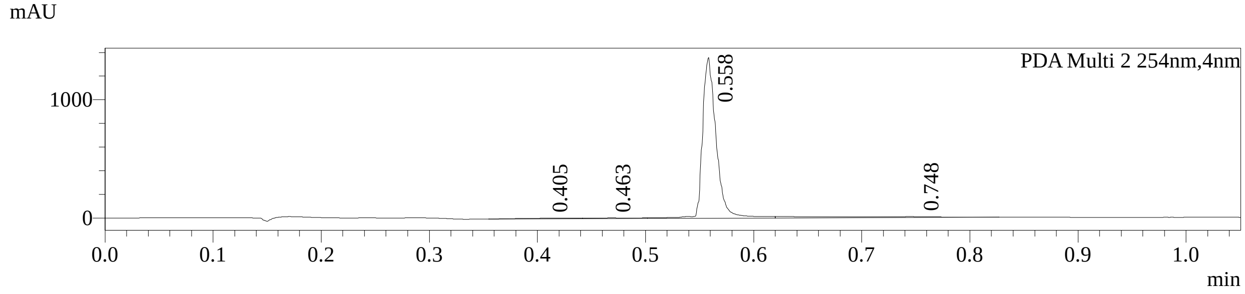

1 PDA Multi 1 / 220nm,4nm
2 PDA Multi 2 / 254nm,4nm

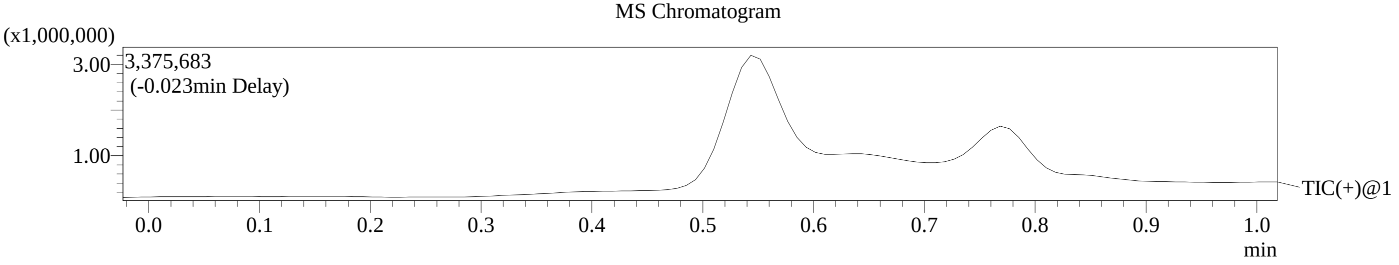

==

Integration Result

==

Peak Table

PDA Ch1 220nm

Peak#	Ret. Time	Height	Height%	USP Width	Area	Area%
1	0.538	78100	1.993	0.027	61447	1.615
2	0.558	3835410	97.858	0.021	3736656	98.223
3	0.677	5863	0.150	0.029	6152	0.162

Peak Table

PDA Ch2 254nm

Peak#	Ret. Time	Height	Height%	USP Width	Area	Area%
1	0.405	5926	0.435	2.085	23647	1.775
2	0.463	6385	0.469	0.974	21283	1.598
3	0.558	1341842	98.548	0.022	1202094	90.241
4	0.748	7466	0.548	0.366	85070	6.386

WEIZ-WX-04-009

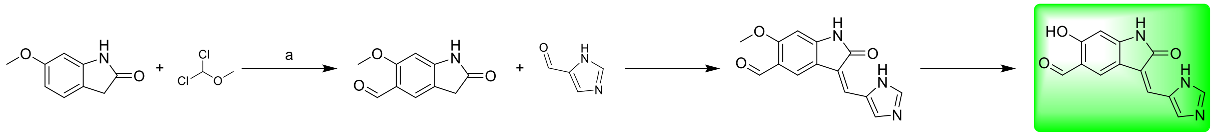

1 2 3 4 5 WEIZ-WX-04-009

Scheme 2: synthetic scheme: (a) $TiCl_4$, DCM, 25 °C, 1 h (b) piperidine alcohol, 90 °C, 12 h (c) BBr_3, DCM, 0 °C, 1 h.

6-methoxy-2-oxo-indoline-5-carbaldehyde

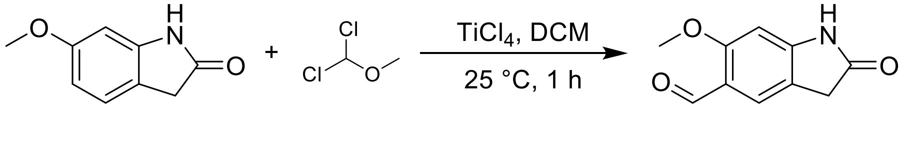

1 2 3

To a solution of 6-methoxyindolin-2-one (500 mg, 1 eq) in dry DCM (15 mL) was added $TiCl_4$ (1.74 g, 1 mL, 3 eq) followed by dichloro(methoxy)methane (528.39 mg, 406.45 µL, 1.5 eq) under 0° C. The mixture was stirred at 25 °C for 1hr. The reaction mixture was quenched with ice water (10 ml). The residue was further purification by pre-HPLC (FA conditions) and lyophilizated to afford a white solid (120 mg, 20.48% yield).

LC-MS (m/z): Calculated: 191.18; Found: 192.0 $[M+H]^+$.

(3Z)-3-(1H-imidazol-5-ylmethylene)-6-methoxy-2-oxo-indoline-5-carbaldehyde:

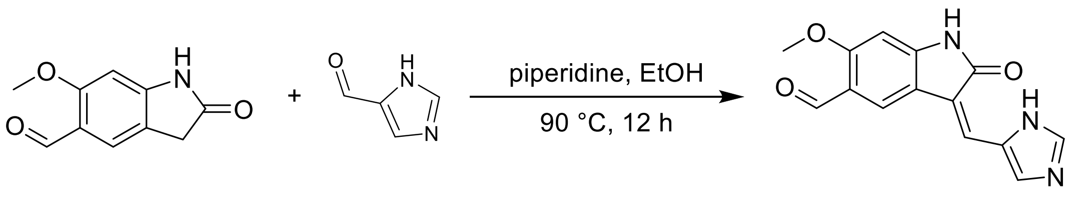

3 4 5

To a solution of 6-methoxy-2-oxo-indoline-5-carbaldehyde (60 mg, 1 eq) and 1H-imidazole-5-carbaldehyde (30.16 mg, 1 eq) in EtOH (2 mL) was added piperidine (2.67 mg, 3.10 µL, 0.1 eq). The mixture was stirred at 90 °C for 12 hr. The reaction mixture was filtered with diatomaceous earth and the filtrate was concentrated under reduced pressure to give a residue. The residue

was further purification by pre-HPLC (FA conditions) and lyophilizated to afford a yellow oil (40 mg, 47.34% yield).

LC-MS (m/z): Calculated: 269.26; Found: 270.1 [M+H] $^+$.

(3Z)-6-hydroxy-3-(1H-imidazol-5-ylmethylene)-2-oxo-indoline-5-carbaldehyde:

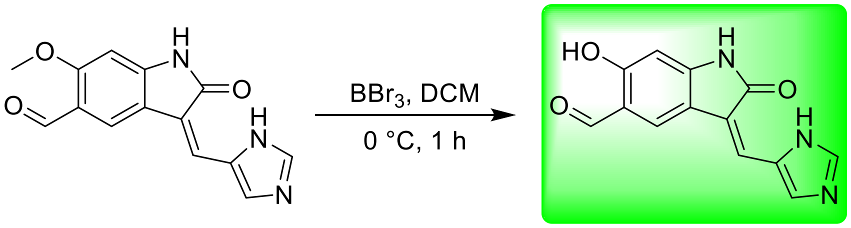

5 **WEIZ-WX-04-009**

To a solution of (3Z) -3-(1H-imidazol -5- ylmethylene) -6-methoxy-2-oxo-indoline-5-carbaldehyde (28.17 mg, 1 eq) in DCM (2 mL) was added BBr_3 (532.57 mg, 204.84 µL, 20.32 eq) under 0 °C. The mixture was stirred at 0 °C for 1 hr. The reaction mixture was quenched with ice water (10 ml). The residue was further purification by prep-HPLC (FA conditions) and lyophilizated to afford a yellow solid (3.83 mg, 14.34% yield).

LC-MS (m/z): Calculated: 255.23; Found: 256.1[M+H] $^+$. Purity: 97%.

^{1}H NMR (400 MHz, DMSO) δ 13.56 - 13.00 (m, 1H), 11.76 - 10.60 (m, 2H), 10.13 (s, 1H), 7.97 (br d, J = 8.0 Hz, 2H), 7.86 - 7.79 (m, 1H), 7.61 (br s, 1H), 6.51 - 6.41 (m, 1H).

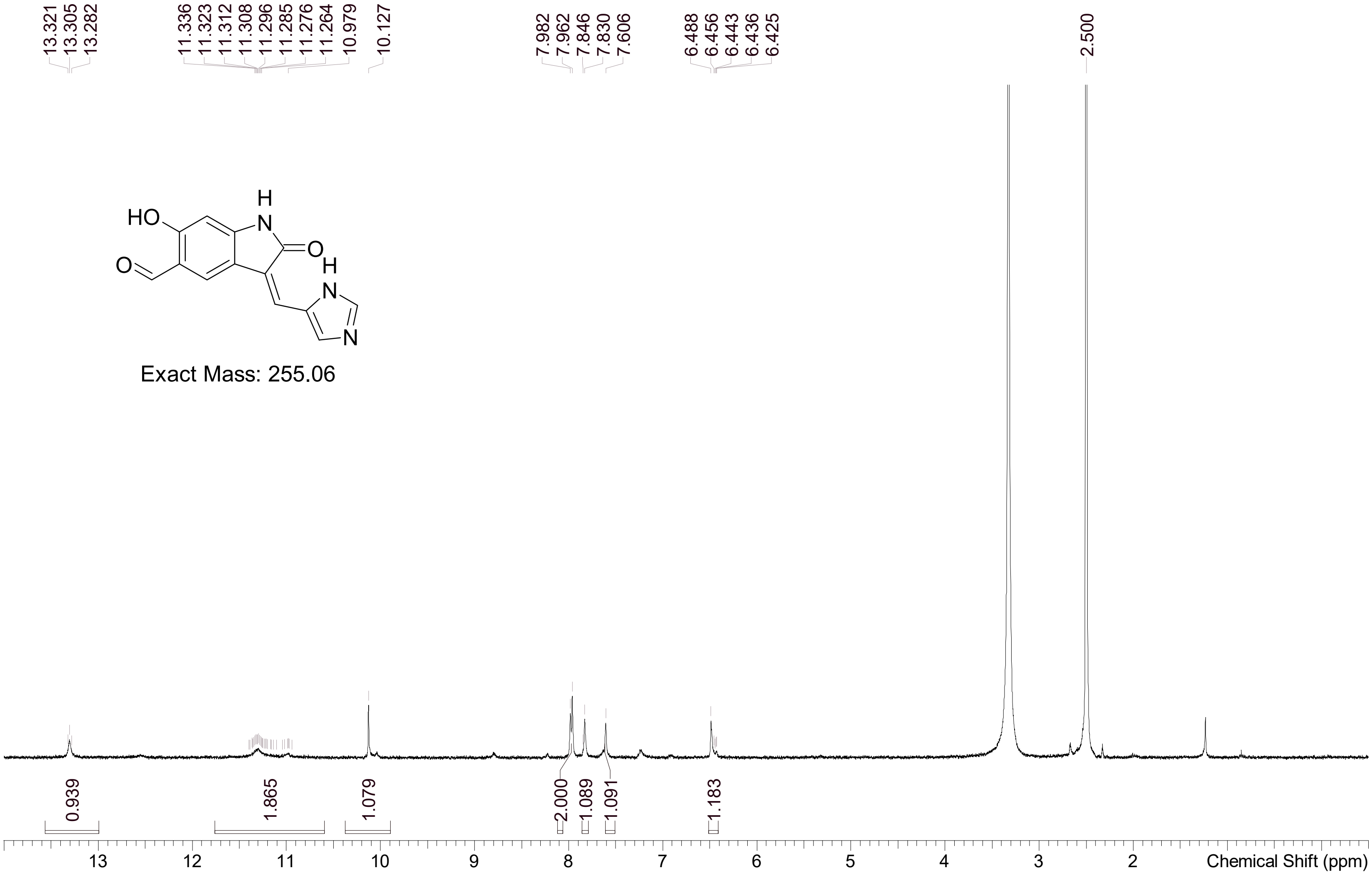

Acquisition Time (sec)	1.9999
Comment	EW44807-463-P1b1 DMSO BRUKER_K_400MHz
Date	21 Nov 2023 10:34:06 (GMT+08:00)
Frequency (MHz)	400.2800
Nucleus	1H
Number of Transients	1
Origin	Avance neo400
Original Points Count	16393
Owner	nmrsu
Points Count	65536
Pulse Sequence	zg
Receiver Gain	18.00
SW(cyclical) (Hz)	8196.72
Solvent	DMSO-d6
Spectrum Offset (Hz)	2467.0876
Spectrum Type	standard
Sweep Width (Hz)	8196.60
Temperature (degree C)	24.100

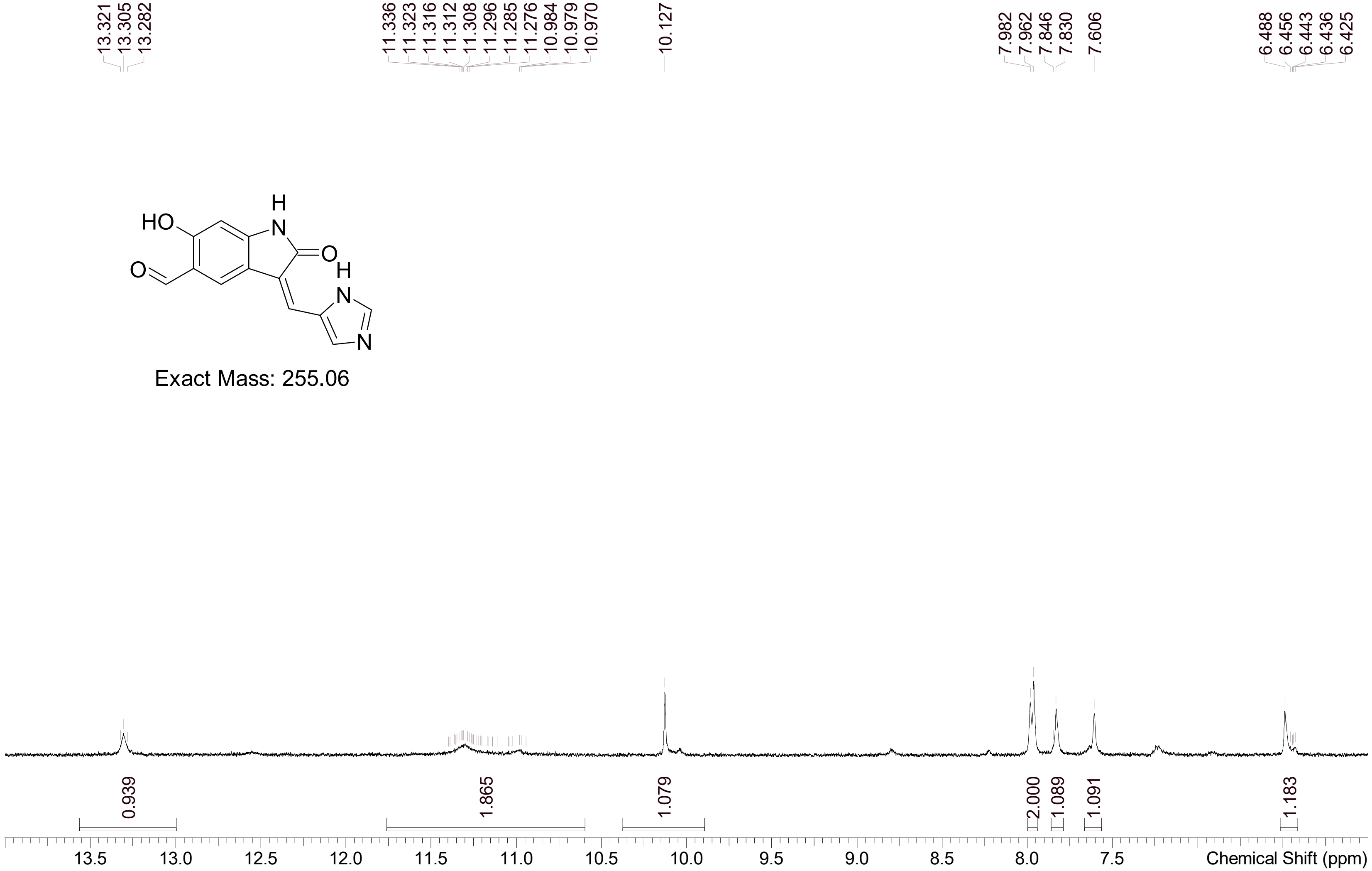

Acquisition Time (sec)	1.9999
Comment	EW44807-463-P1b1 DMSO BRUKER_K_400MHz
Date	21 Nov 2023 10:34:06 (GMT+08:00)
Frequency (MHz)	400.2800
Nucleus	1H
Number of Transients	1
Origin	Avance neo400
Original Points Count	16393
Owner	nmrsu
Points Count	65536
Pulse Sequence	zg
Receiver Gain	18.00
SW(cyclical) (Hz)	8196.72
Solvent	DMSO-d6
Spectrum Offset (Hz)	2467.0876
Spectrum Type	standard
Sweep Width (Hz)	8196.60
Temperature (degree C)	24.100

Chromatogram

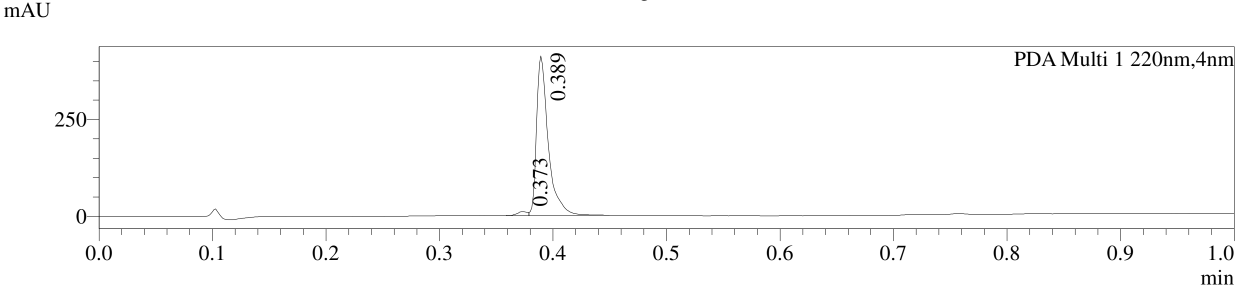

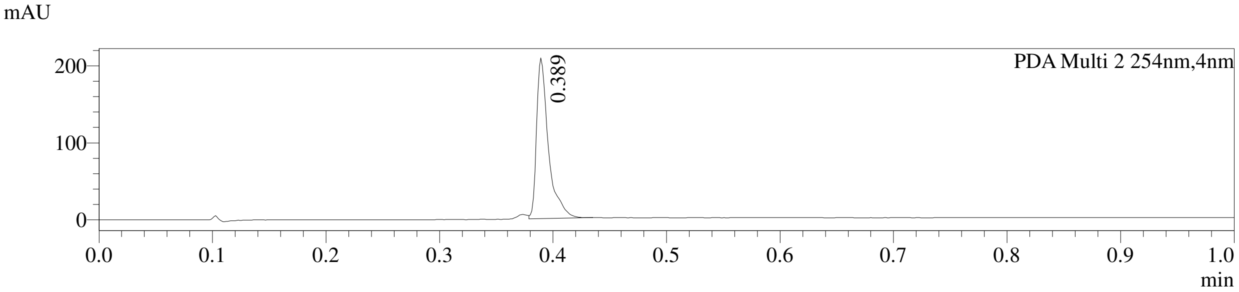

1 PDA Multi 1 / 220nm,4nm
2 PDA Multi 2 / 254nm,4nm

MS Chromatogram

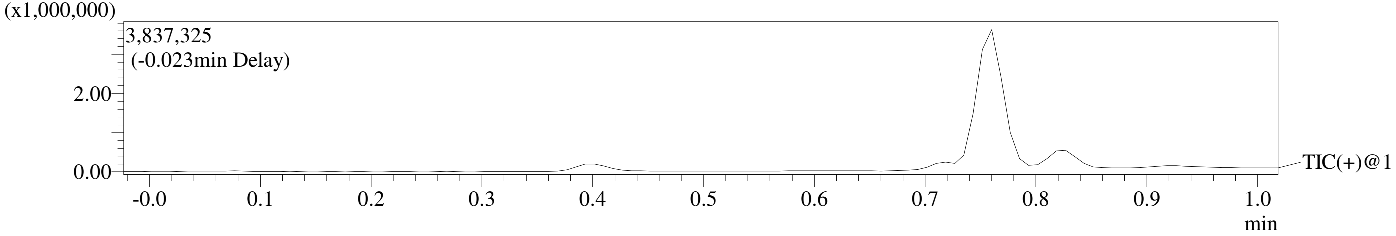

==
Integration Result
==

Peak Table

PDA Ch1 220nm

Peak#	Ret. Time	Height	Height%	USP Width	Area	Area%
1	0.373	10285	2.436	0.032	6730	2.223
2	0.389	411857	97.564	0.020	296063	97.777

Peak Table

PDA Ch2 254nm

Peak#	Ret. Time	Height	Height%	USP Width	Area	Area%
1	0.389	208711	100.000	0.020	151947	100.000

WEIZ-WX-04-010

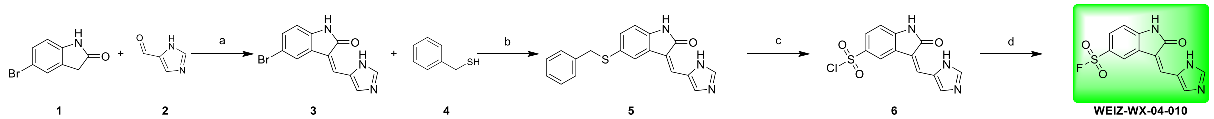

Scheme 3: synthetic scheme: (a) piperidine, EtOH, 90 °C, 12 h (b) $Pd_2(dba)_3$, Xantphos, DIEA, dioxane, 100 °C, 12 h (c) NCS, HOAc/H_2O, 0-25 °C, 2 h (d) KF, 18-crown-6 ether, ACN, 20 °C, 2 h

(3Z)-5-bromo-3-(1H-imidazol-5-ylmethylene)indolin-2-one:

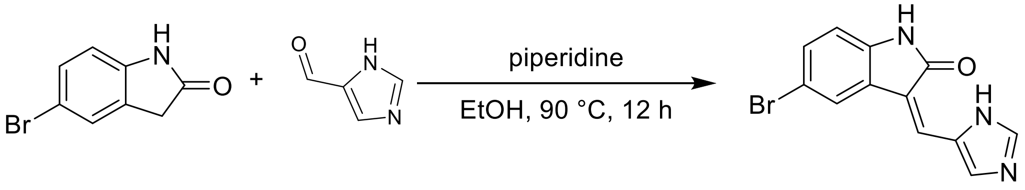

1 2 3

To a solution of 5-bromoindolin-2-one (1 g, 1 eq) and 1H-imidazole-5-carbaldehyde (543.78 mg, 1.2 eq) in EtOH (10 mL) was added piperidine (40.16 mg, 46.57 µL, 0.1 eq). The mixture was stirred at 90 °C for 12 hr. The reaction mixture was concentrated under reduced pressure to give a residue. The residue was further purification by prep-HPLC and Lyophilizated to afford a yellow oil (2 g, crude).

Calculated: 290.09; Found: 291.0$[M+H]^+$.

^{1}H NMR (400 MHz, DMSO) δ 13.63 (br d, J = 1.8 Hz, 1H), 11.22 - 11.00 (m, 1H), 8.02 (d, J = 18.0 Hz, 2H), 7.92 (d, J = 1.6 Hz, 1H), 7.64 (s, 1H), 7.41 - 7.27 (m, 1H), 6.85 (d, J = 8.4 Hz, 1H)

(3Z)-5-benzylsulfanyl-3-(1H-imidazol-5-ylmethylene)indolin-2-one:

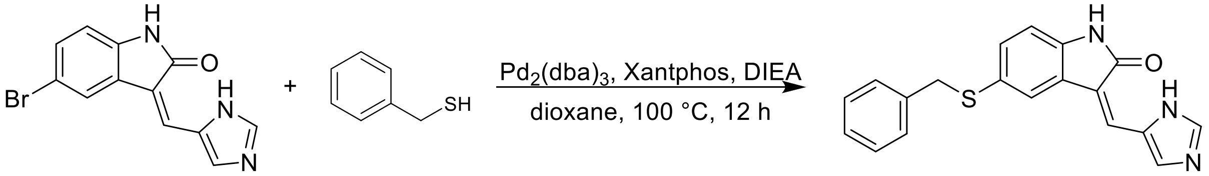

3 4 5

To a solution of (3Z)-5-bromo-3-(1H-imidazol-5-ylmethylene)indolin-2-one (1.4 g, 1 eq) and phenylmethanethiol (2 g, 1.89 mL, 3.34 eq) in dioxane (10 mL) was added DIEA (1.87 g, 2.52 mL,

3 eq), Xantphos (837.67 mg, 0.3 eq) and $Pd_2(dba)_3$ (883.79 mg, 0.2 eq). The mixture was stirred at 100 °C for 12 hr under N_2. The reaction mixture was quenched with $NaClO_4$ (100 ml). The reaction mixture was concentrated under reduced pressure to give a residue. The residue was further purification by pre-HPLC and lyophilizated to afford a yellow solid (400 mg, crude).

LC-MS (m/z): Calculated: 333.41; Found: 334.1[M+H] $^+$.

^{1}H NMR (400 MHz, DMSO) δ 4.14 - 4.21 (m, 2 H) 6.73 (d, J=8.00 Hz, 1 H) 7.12 - 7.34 (m, 7 H) 7.49 (s, 1 H) 7.94 (s, 1 H) 8.07 (s, 1 H) 9.50 (s, 1 H)

(3Z)-3-(1H-imidazol-5-ylmethylene)-2-oxoindoline-5-sulfonyl chloride:

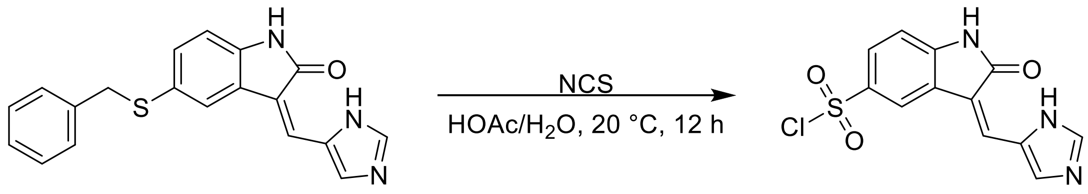

5 6

To a solution of (3Z)-5-benzylsulfanyl-3-(1H-imidazol-5-ylmethylene)indolin-2-one (200 mg, 1 eq) in HOAc (10 mL) and H_2O (2.5 mL) was added NCS (240.31 mg, 1.80 mmol, 3 eq) at 0 °C. The mixture was stirred at 25 °C for 2 hr. The reaction mixture was concentrated under reduced pressure to give a residue to afford a yellow oil (180 mg, 96.88% yield).

LC-MS (m/z): Calculated: 309.33; Found: 308.1 [M-H] $^+$.

(3Z)-3-(1H-imidazol-5-ylmethylene)-2-oxoindoline-5-sulfonyl fluoride:

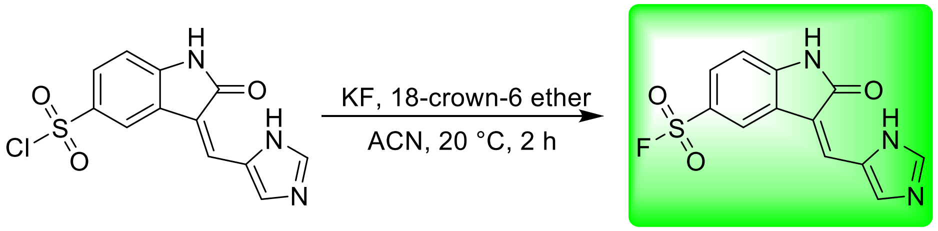

6 **WEIZ-WX-04-010**

To a solution of (3Z)-3-(1H-imidazol-5-ylmethylene)-2-oxo-indoline-5-sulfonylchloride (135 mg, 1 eq) in ACN (2 mL) was added KF (126.61 mg, 5 eq) and 1,4,7,10,13,16-

hexaoxacyclooctadecane (11.52 mg, 0.1 eq). The mixture was stirred at 20 °C for 2 hr under N_2. The residue was further purification by prep-HPLC and lyophilizated to afford a yellow solid (1.45 mg, 1.13% yield, 100% purity).

LC-MS (m/z): Calculated: 293.27; Found: 294.1 $[M+H]^+$. Purity: >95%.

^{1}H NMR (400 MHz, DMSO) δ 10.24 (d, J = 2 Hz, 1H), 8.12 (d, J = 18 Hz, 2H), 7.94 (dd, J = 2, 8. Hz, 1H), 7.70 (s, 1H), 7.14 (d, J = 8.4 Hz, 1H).

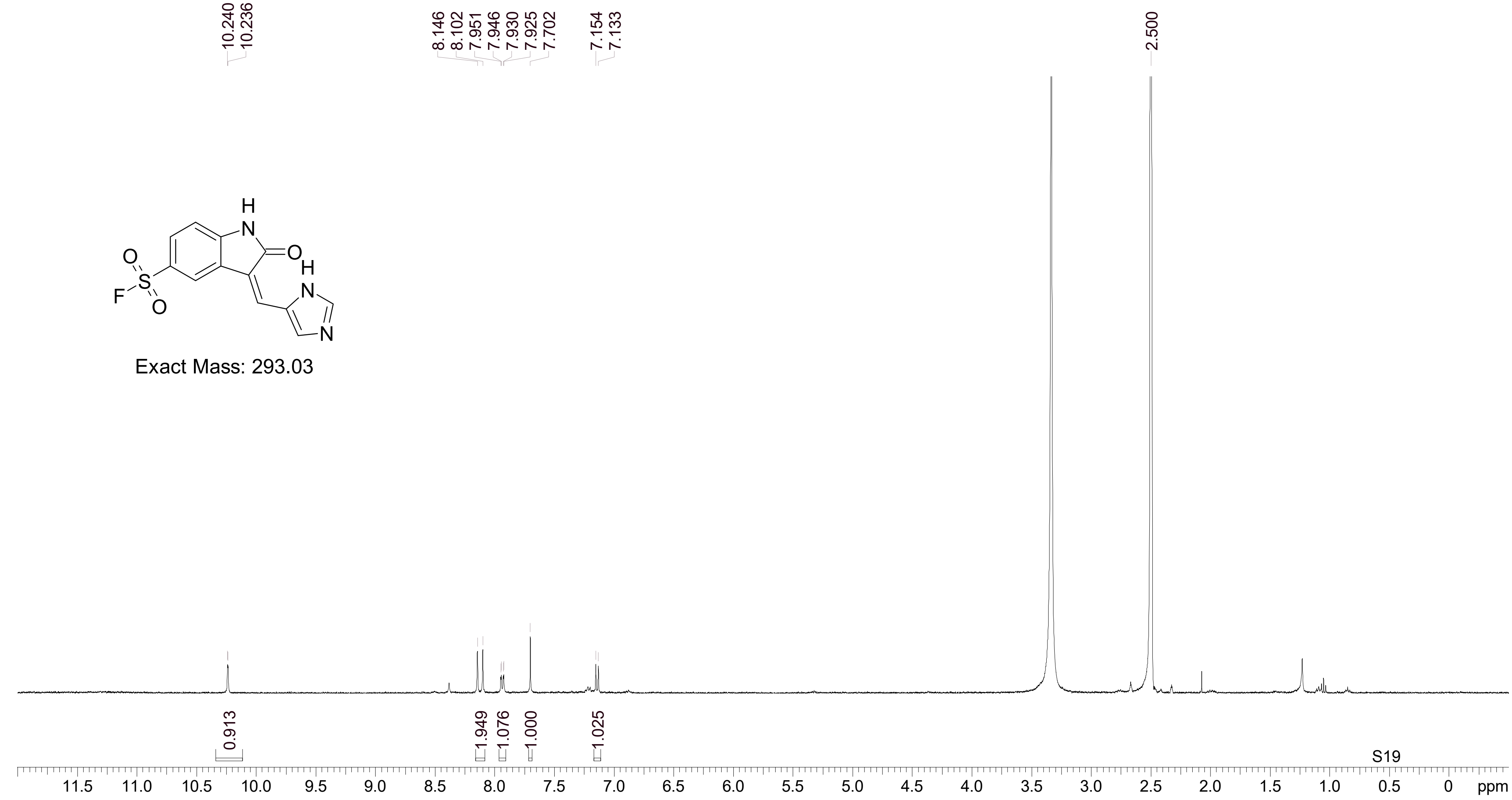
10.240
10.236
8.146
8.102
7.951
7.946
7.930
7.925
7.702
7.154
7.133
2.500
Exact Mass: 293.03
0.913
1.949
1.076
1.000
1.025
11.5 11.0 10.5 10.0 9.5 9.0 8.5 8.0 7.5 7.0 6.5 6.0 5.5 5.0 4.5 4.0 3.5 3.0 2.5 2.0 1.5 1.0 0.5 0 ppm

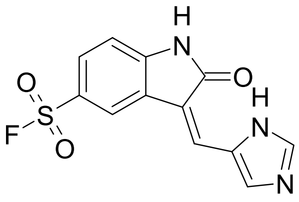

Exact Mass: 293.03

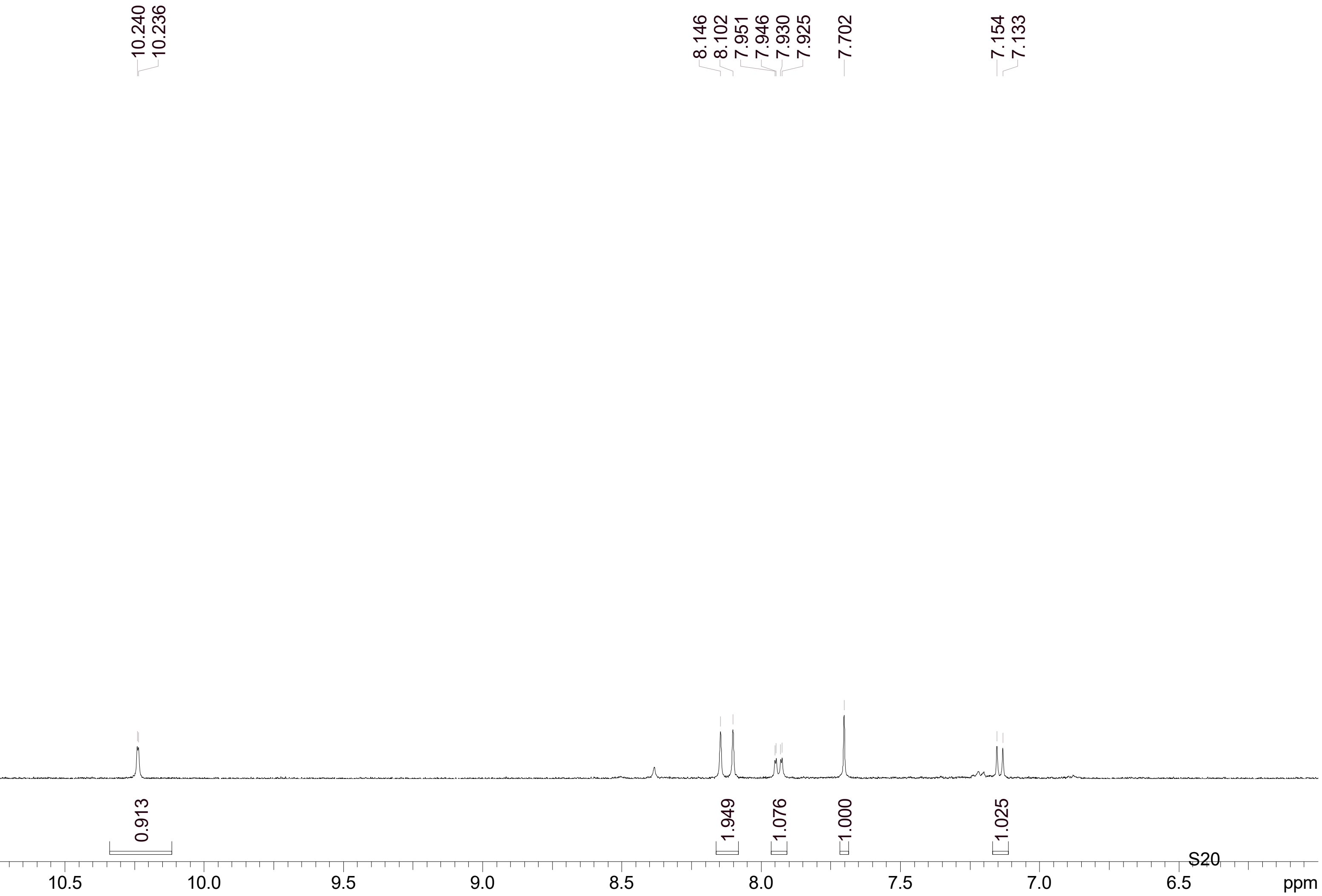

Chromatogram

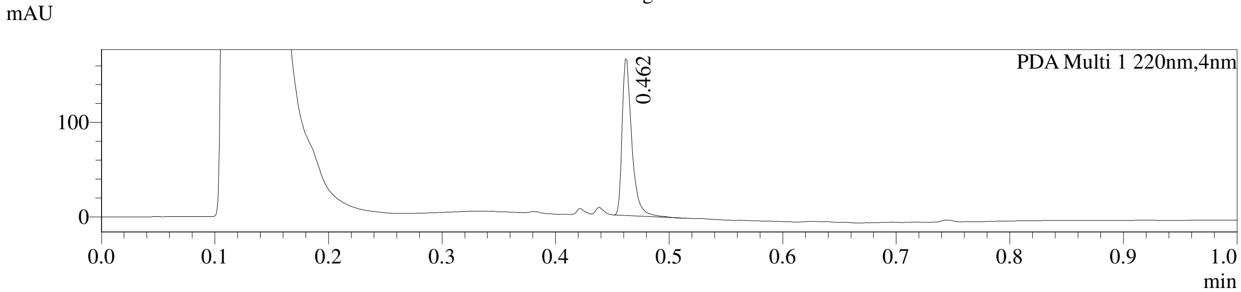

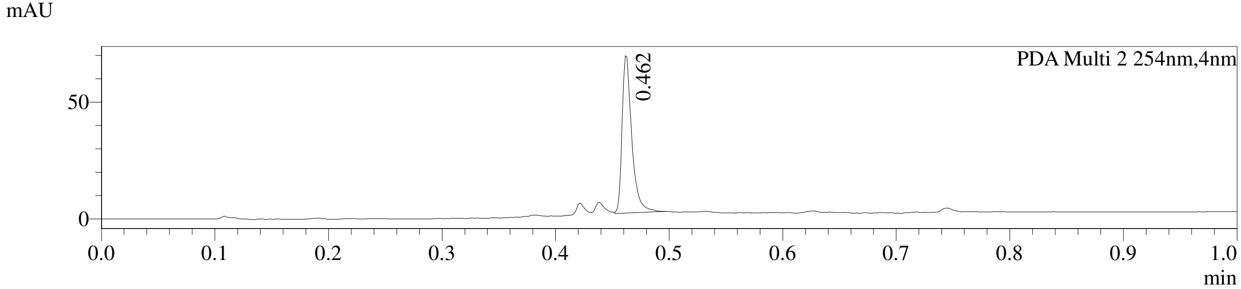

1 PDA Multi 1 / 220nm,4nm
2 PDA Multi 2 / 254nm,4nm

MS Chromatogram

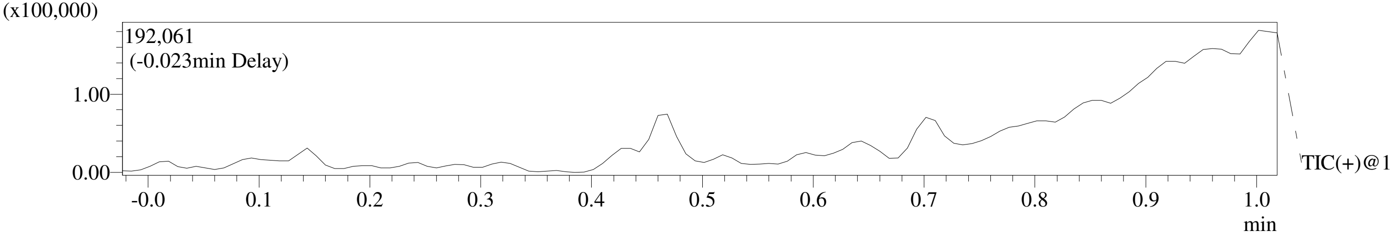

Integration Result

Peak Table

PDA Ch1 220nm

Peak#	Ret. Time	Height	Height%	USP Width	Area	Area%
1	0.462	165882	100.000	0.017	98525	100.000

Peak Table

PDA Ch2 254nm

Peak#	Ret. Time	Height	Height%	USP Width	Area	Area%
1	0.462	67329	100.000	0.017	40100	100.000

S21

WEIZ-WX-04-011

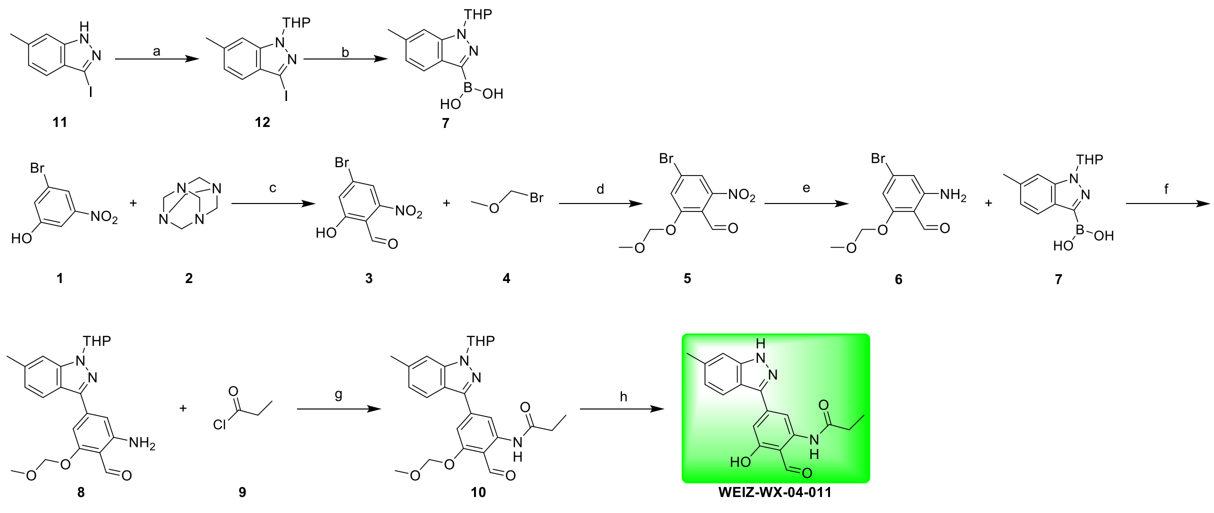

Scheme 4: synthetic scheme: (a) DHP, PTSA, EtOAc, 80 °C, 16 h (b) B_2pin_2, Pd(dppf)Cl_2, KOAc, dioxane, 100 °C (c) TFA, 120 °C, 12 h (d) DIEA, DCM, 25 °C, 12 h (e) Fe, NH_4Cl, EtOH, H_2O, 80 °C, 1 h (f) Pd(dppf)Cl_2, Na_2CO_3, K_2CO_3, dioxane/H_2O, 90 °C, 12 h (g) TEA, DCM, 0 °C, 0.5 h (h) TFA, 25 °C, 1 h.

3-iodo-6-methyl-1-tetrahydropyran-2-ylindazole:

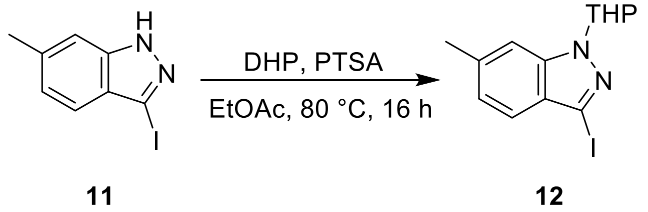

To a solution of 3-iodo-6-methyl-1H-indazole (1.2 g, 1 eq) and PTSA (160.15 mg, 0.2 eq) in EtOAc (10 mL) was added 3,4-dihydro-2H-pyran (782.30 mg, 850.32 µL, 2 eq). The mixture was stirred at 80 °C for 16 hr under N_2. The reaction was purified by prep-TLC (Petroleum ether/Ethyl acetate = 2/1) to afford a yellow oil (800 mg, 50.28% yield).

LC-MS (m/z): Calculated: 342.17; Found: 343.0 $[M+H]^+$.

^{1}H NMR (400 MHz, DMSO) δ 7.54 (s, 1H), 7.31 (d, J = 8.4 Hz, 1H), 7.10 (dd, J = 0.6, 8.4 Hz, 1H), 5.79 (dd, J = 2.4, 9.8 Hz, 1H), 3.93 - 3.81 (m, 1H), 3.77 - 3.66 (m, 1H), 2.47 (s, 3H), 2.42 - 2.29 (m, 1H), 2.08 - 1.89 (m, 2H), 1.80 - 1.66 (m, 1H), 1.63 - 1.51 (m, 2H).

(6-methyl-1-tetrahydropyran-2-yl-indazol-3-yl)boronic acid:

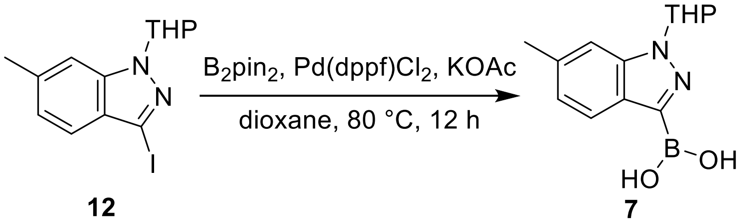

12 7

A mixture of 3-iodo-6-methyl-1-tetrahydropyran-2-yl-indazole (200 mg, 1 eq), 4,4,5,5-tetramethyl-2-(4,4,5,5-tetramethyl-1,3,2-dioxaborolan-2-yl) -1,3,2-dioxaborolane (296.85 mg, 2 eq) and KOAc (143.41 mg, 2.5 eq) in dioxane (4 mL) was added cyclopentyl (diphenyl) phosphane;dichloromethane;dichloropalladium;iron (47.73 mg, 0.1 eq) then degassed and purged with N_2 for 3 times, the reaction mixture was stirred at 80 °C for 12hr under N_2 atmosphere. The reaction mixture was filtered with diatomaceous earth and the filtrate was concentrated under reduced pressure to give a residue. The residue was further purification by pre-HPLC (FA conditions) and lyophilizated to afford a yellow oil (100 mg, 65.78% yield).

LC-MS (m/z): Calculated: 260.1; Found: 261.1$[M+H]^+$.

^1H NMR (400 MHz, DMSO) δ 8.01 (s, 1H), 7.01 (d, J = 7.8 Hz, 1H), 5.78 (dd, J = 2.4, 9.8 Hz, 1H), 4.01 - 3.62 (m, 2H), 2.45 (s, 3H), 2.44 - 2.29 (m, 2H), 2.10 - 1.88 (m, 2H), 1.83 - 1.47 (m, 3H).

4-bromo-2-hydroxy-6-nitro-benzaldehyde:

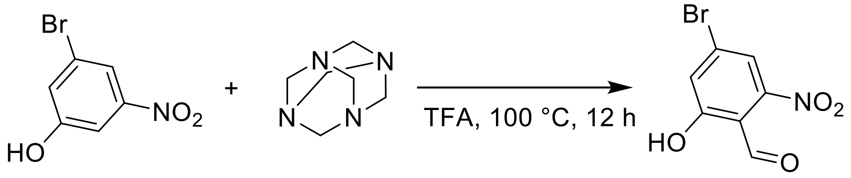

1 2 3

To a solution of 3-methylcyclohex-2-en-1-one (10 g, 1 *eq*), methyl 2-cyanoacetate (25.64 g, 2.85 *eq*) in Tol. (100 mL) was added CH_3COOH (16.30 g, 2.99 *eq*) and CH_3COONH_4 (17.84 g, 2.55 *eq*). The mixture was stirred at 60 °C for 12 hr. The reaction mixture was poured into water (200 ml), extracted with Ethyl acetate (100 mL × 3). The combined organic layers were washed

with brine (100 mL), dried over Na_2SO_4, filtered and concentrated under reduced pressure to give a residue. The residue was purified by pre-HPLC (FA conditions) to afford a yellow oil (12 g, 69.12% yield).

LC-MS (m/z): Calculated: 191.09; Found: 192.0 [M+H] $^+$.

^{1}H NMR (400 MHz, DMSO) δ 7.65 - 6.47 (m, 1H), 3.85 - 3.72 (m, 3H), 3.06 - 2.67 (m, 2H), 2.32 (d, *J* = 7.2 Hz, 2H), 2.05 (d, *J* = 12.6 Hz, 3H), 1.89 - 1.70 (m, 2H)

4-bromo-2-(methoxymethoxy)-6-nitro-benzaldehyde:

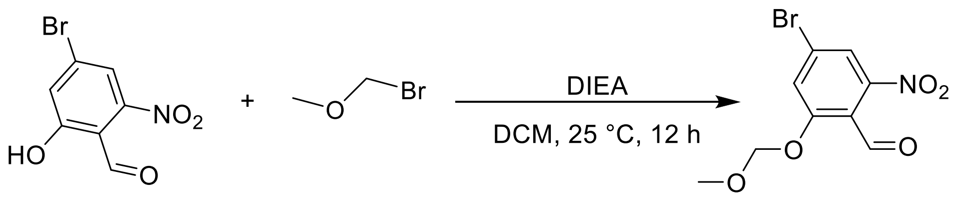

3 **4** **5**

To a solution of 4-bromo-2-hydroxy-6-nitro-benzaldehyde (4 g, 1 eq) in DCM (60 mL) was added DIEA (3.15 g, 4.25 mL, 1.5 eq) and bromo(methoxy)methane (3.05 g, 1.99 mL, 1.5 eq) at 0 °C. The mixture was stirred at 0-25 °C for 12 hr. The reaction mixture was concentrated under vacuum to give the residue to afford a yellow solid (5.3 g, crude).

LC-MS (m/z): Calculated: 289.09; Found: 287.7 [M-H] $^+$.

2-amino-4-bromo-6-(methoxymethoxy)benzaldehyde:

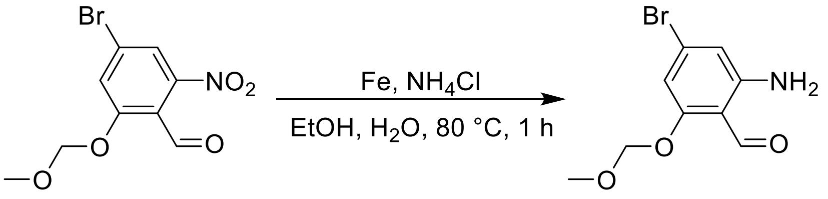

5 **6**

To a solution of 4-bromo-2-(methoxymethoxy)-6-nitro-benzaldehyde (5.2 g, 1 eq) , NH_4Cl (9.59 g, 10 eq) in EtOH (100 mL) and H_2O (30 mL) was added Fe (5.01 g, 5 eq). The mixture was stirred at 80 °C for 1 hr. The reaction mixture was filtered and the filtrate was concentrated under

vacuum to give the residue. The residue was purified by prep-HPLC and lyophilized to afford a yellow solid (490 mg, 10.51% yield).

LC-MS (m/z): Calculated: 257.09; Found: 258.1 $[M+H]^+$.

^1H NMR (400 MHz, DMSO) δ 10.30 (s, 1H), 7.83 - 7.35 (m, 2H), 6.66 (d, J = 1.4 Hz, 1H), 6.45 (d, J = 1.6 Hz, 1H), 5.32 (s, 2H), 3.47 - 3.45 (m, 3H).

2-amino-6-(methoxymethoxy)-4-(6-methyl-1-tetrahydropyran-2-yl-indazol-3-yl)benzaldehyde:

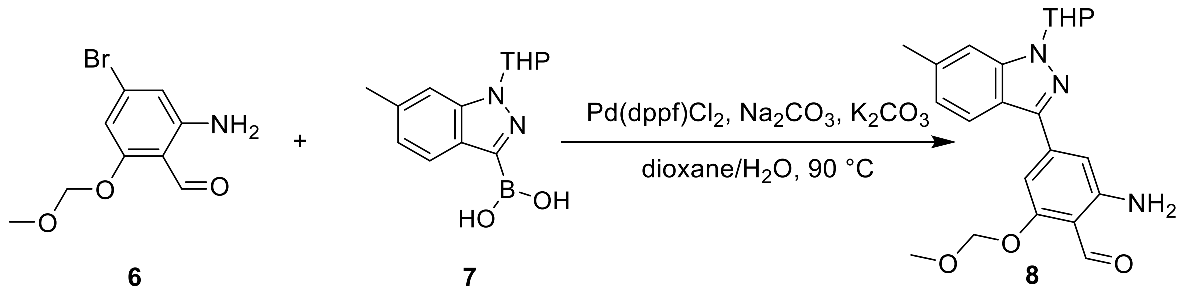

A mixture of 2-amino-4-bromo-6-(methoxymethoxy)benzaldehyde (50 mg, 1 eq), (6-methyl-1-tetrahydropyran-2-yl-indazol-3-yl)boronic acid (60.00 mg, 1.2 eq), Na_2CO_3 (61.13 mg, 3 eq) and K_2CO_3 (79.71 mg, 3 eq) in dioxane (4 mL) and H_2O (1 mL) was added $Pd(dppf)Cl_2.CH_2Cl_2$ (15.70 mg, 0.1 eq) then degassed and purged with N_2 for 3 times, the reaction mixture was stirred at 90 ° C for 12 hr under N_2 atmosphere. The reaction mixture was filtered and the filtrate was concentrated under vacuum to give the residue. The residue was purified by prep-HPLC and lyophilized to afford as a brown solid (20 mg, 26.31% yield).

LC-MS (m/z): Calculated: 395.45; Found: 396.2 $[M+H]^+$.

^1H NMR (400 MHz, DMSO) δ 10.43 (s, 1H), 7.95 (d, J = 8.6 Hz, 1H), 7.54 (s, 1H), 7.17 (d, J = 8.4 Hz, 1H), 7.06 (s, 1H), 6.99 (d, J = 1.2 Hz, 1H), 5.87 (dd, J = 2.5, 9.6 Hz, 1H), 5.40 (s, 2H), 4.12 - 4.01 (m, 1H), 3.93 - 3.82 (m, 1H), 3.58 (s, 3H), 2.55 (s, 3H), 2.25 - 2.15 (m, 1H), 2.12 - 2.05 (m, 1H), 1.97 - 1.80 (m, 2H), 1.79 - 1.65 (m, 2H)

N-[2-formyl-3-(methoxymethoxy)-5-(6-methyl-1-tetrahydropyran-2-ylindazol-3-yl)phenyl]propanamide:

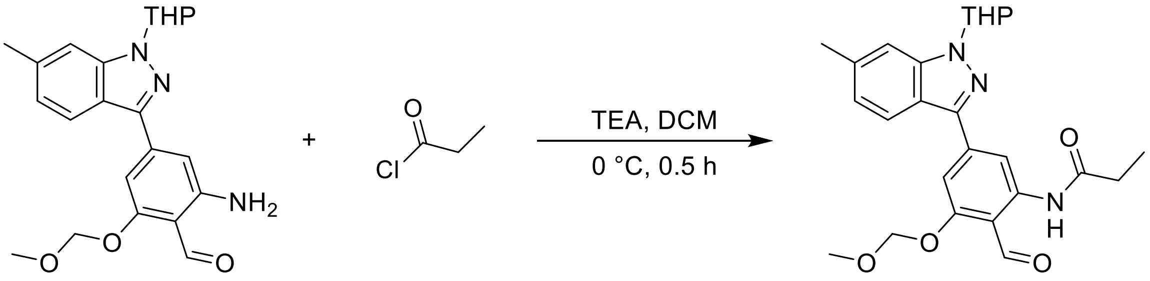

8 **9** **10**

To a solution of 2-amino-6-(methoxymethoxy)-4-(6-methyl-1-tetrahydropyran-2-yl-indazol-3-yl)benzaldehyde (15 mg, 1 eq) in DCM (1 mL) was added TEA (11.51 mg, 15.84 µL, 3 eq) and propanoylchloride (7.02 mg, 7.02 µL, 2 eq). The mixture was stirred at 0 °C for 0.5 hr. The reaction mixture was concentrated under vacuum to give the residue to afford a yellow solid (15 mg, crude).

LC-MS (m/z): Calculated: 451.1; Found: 450.1 [M-H] $^{+}$.

N-[2-formyl-3-hydroxy-5-(6-methyl-1H-indazol-3-yl)phenyl]propanamide:

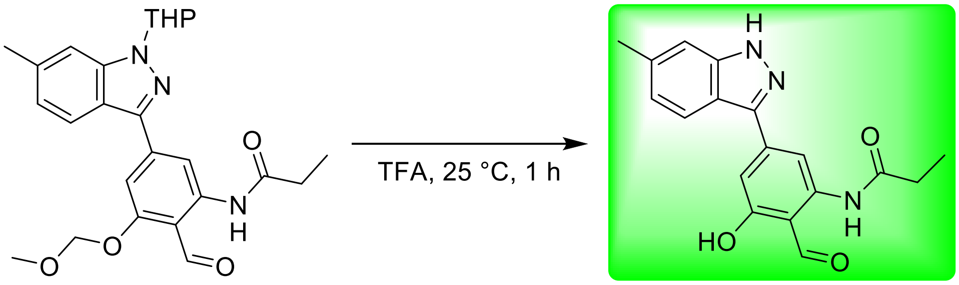

10 **WEIZ-WX-04-011**

To a solution of N-[2-formyl-3-(methoxymethoxy) -5-(6-methyl-1-tetrahydropyran-2-yl-indazol-3-yl) phenyl] propanamide (15 mg, 1 eq) in TFA (1 mL). The mixture was stirred at 25 °C for 1 hr. The reaction mixture was concentrated under vacuum to give the residue. The residue was purified by prep-HPLC and lyophilized to afford a yellow solid (0.53 mg, 4.59% yield, 93% purity).

LC-MS (m/z): Calculated: 323.35; Found: 324.0 [M+H] $^{+}$. Purity: >95%.

^{1}H NMR (400 MHz, DMSO) δ 13.38 - 13.25 (m, 1H), 11.56 (br d, J = 3.8 Hz, 1H), 10.33 (s, 1H), 8.70 - 8.55 (m, 1H), 7.95 (d, J = 9.0 Hz, 1H), 7.39 (s, 1H), 7.34 - 7.24 (m, 1H), 7.12 (d, J = 7.8 Hz, 1H), 2.47 (s, 5H), 1.16 (t, J = 7.6 Hz, 3H).

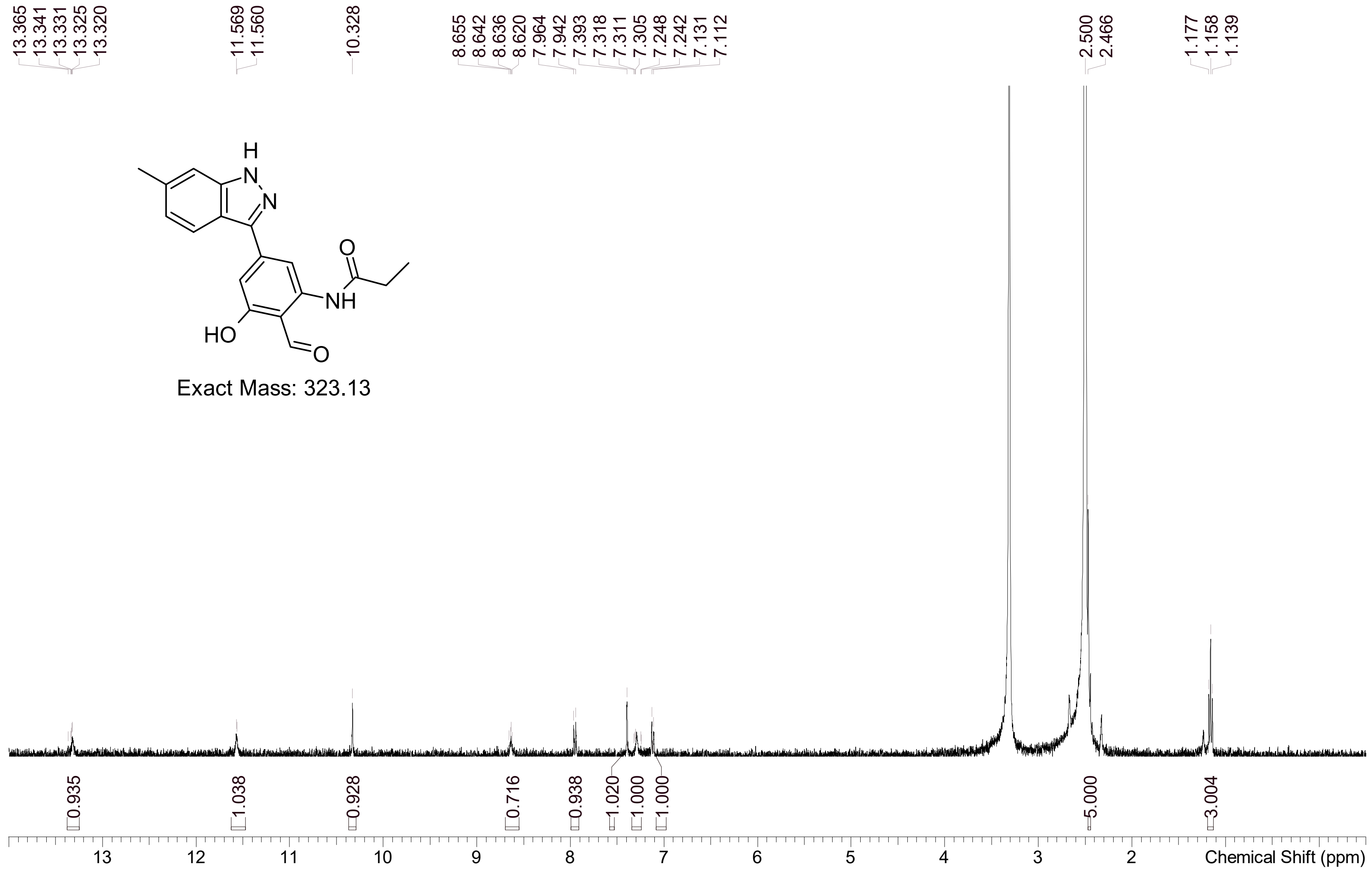

Acquisition Time (sec)	1.9999
Comment	EW43877-457-P1A DMSO BRUKER_O_400MHz
Date	25 Jan 2024 11:12:05 (GMT+08:00)
Frequency (MHz)	400.1300
Nucleus	1H
Number of Transients	1
Origin	Avance
Original Points Count	16393
Owner	nmrsu
Points Count	65536
Pulse Sequence	zg
Receiver Gain	18.00
SW(cyclical) (Hz)	8196.72
Solvent	DMSO-d6
Spectrum Offset (Hz)	2468.0007
Spectrum Type	standard
Sweep Width (Hz)	8196.60
Temperature (degree C)	26.077

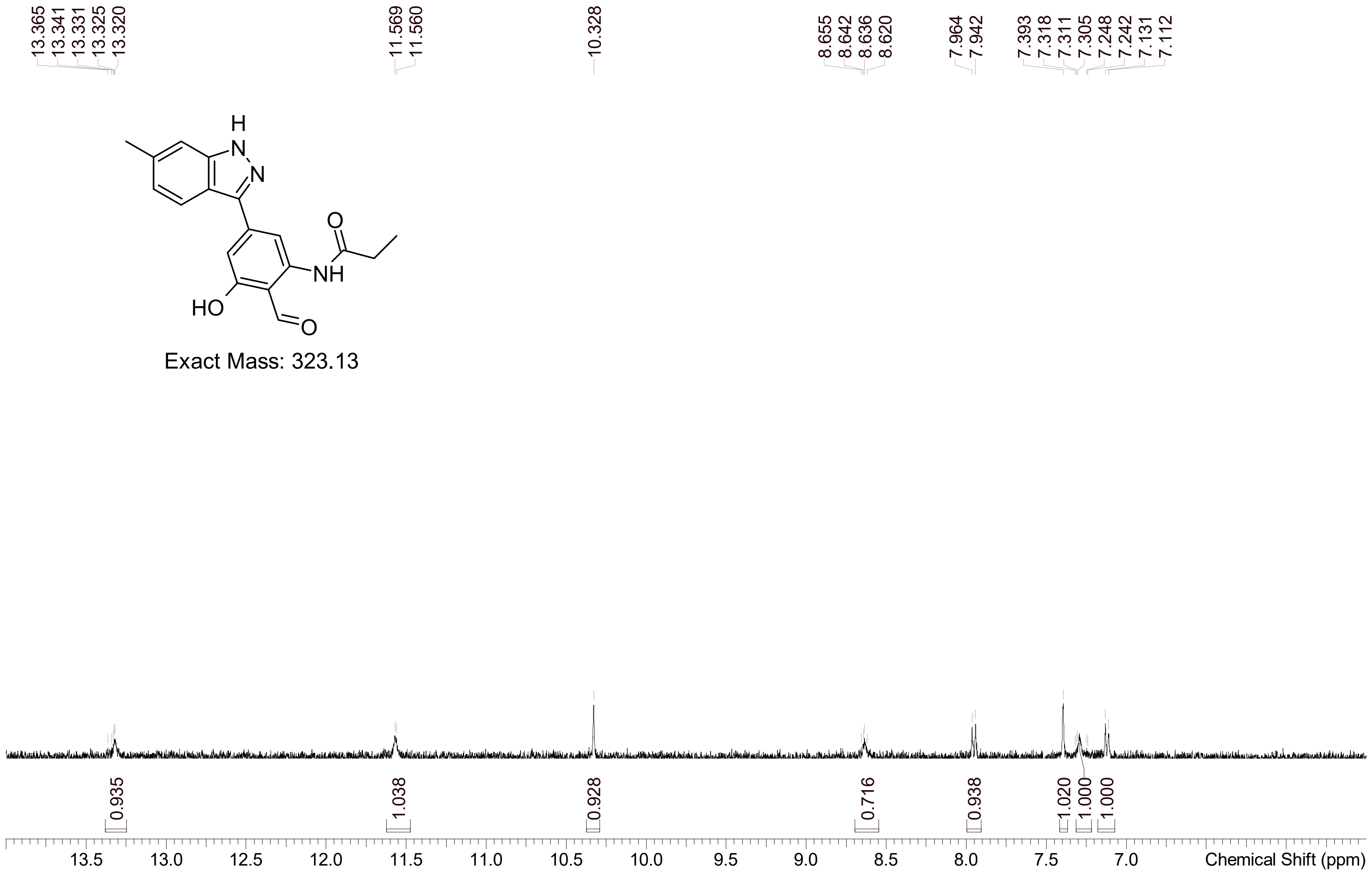

Acquisition Time (sec)	1.9999
Comment	EW43877-457-P1A DMSO BRUKER_O_400MHz
Date	25 Jan 2024 11:12:05 (GMT+08:00)
Frequency (MHz)	400.1300
Nucleus	1H
Number of Transients	1
Origin	Avance
Original Points Count	16393
Owner	nmrsu
Points Count	65536
Pulse Sequence	zg
Receiver Gain	18.00
SW(cyclical) (Hz)	8196.72
Solvent	DMSO-d6
Spectrum Offset (Hz)	2468.0007
Spectrum Type	standard
Sweep Width (Hz)	8196.60
Temperature (degree C)	26.077

Chromatogram

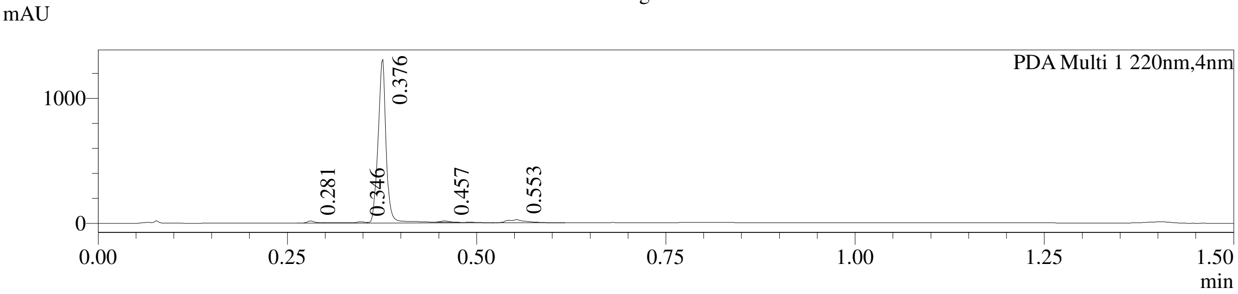

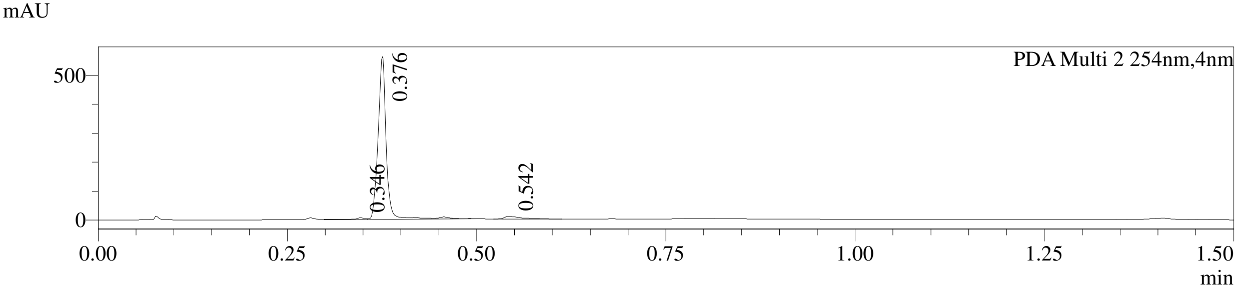

1 PDA Multi 1 / 220nm,4nm
2 PDA Multi 2 / 254nm,4nm

MS Chromatogram

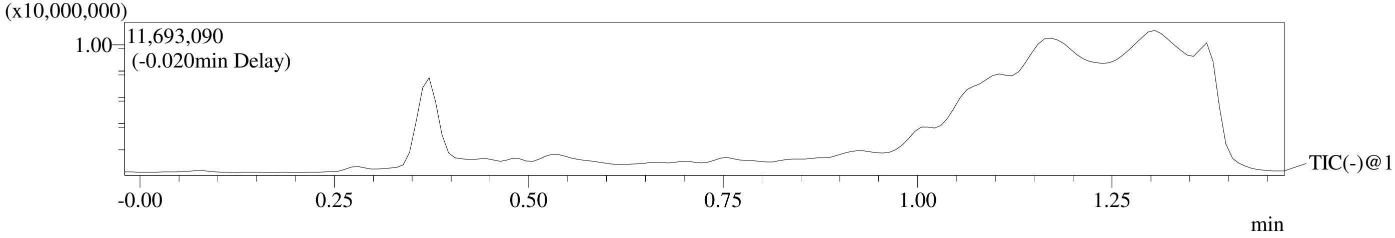

Integration Result

Peak Table

PDA Ch1 220nm

Peak#	Ret. Time	Height	Height%	USP Width	Area	Area%
1	0.281	16300	1.188	0.021	11730	1.112
2	0.346	10076	0.735	0.023	10450	0.991
3	0.376	1309989	95.501	0.021	982062	93.097
4	0.457	10099	0.736	0.023	7799	0.739
5	0.553	25233	1.840	0.038	42839	4.061

Peak Table

PDA Ch2 254nm

Peak#	Ret. Time	Height	Height%	USP Width	Area	Area%
1	0.346	5906	1.019	0.023	5967	1.348
2	0.376	564323	97.394	0.021	421754	95.246
3	0.542	9195	1.587	0.038	15082	3.406
